# Discovery and Optimization of Novel 5-Indolyl-7-arylimidazo[1,2-*a*]pyridine-8-carbonitrile Derivatives as Potent Antitubulin Agents Targeting Colchicine-binding Site

**DOI:** 10.1038/srep43398

**Published:** 2017-02-27

**Authors:** Xin Zhai, Xiaoqiang Wang, Jiao Wang, Jin Liu, Daiying Zuo, Nan Jiang, Tianfang Zeng, Xiuxiu Yang, Tongfei Jing, Ping Gong

**Affiliations:** 1 Key Laboratory of Structure-Based Drug Design and Discovery, Ministry of Education, Shenyang Pharmaceutical University, 103 Wenhua Road, Shenhe District, Shenyang, 110016, China; 2 Department of Pharmacology, Shenyang Pharmaceutical University, 103 Wenhua Road, Shenhe District, Shenyang, 110016, China

## Abstract

Aiming at development of potent antitubulin agents targeting colchicine-binding site, a series of novel 5-indolyl-7-arylimidazo[1,2-*a*]pyridine-8-carbonitrilederivatives (5a–5v and 7a–7h) were designed based on bioisosterism and hybridization strategies. All these compounds were concisely synthesized *via* a three-step process and examined against five human cancer cell lines (HT-29, A549, MKN-45, MDA-MB-231 and SMMC-7721) along with a normal human cell (L02) *in vitro*. A structure-activity relationships (SARs) study was carried out and optimization towards this series of compounds in cellular assay resulted in the discovery of **5k**, which displayed similar or better antitumor potency against the tested cancer cells with IC_50_ value ranging from 0.02 to 1.22 μM superior to CA-4 and Crolibulin. Significantly, a cell cycle study disclosed the ability of **5k** to arrest cell cycle at the G2/M phase, and immunofluorescence assay as well as a colchicine competition assay revealed that tubulin polymerization was disturbed by **5k** by binding to the colchicine site. Moreover, the molecular modeling mode showed the posture of **5k** and Crolibulin was similar in the colchcine-binding pocket of tubulin as identified with the SARs and pharmacological results. Together, all these results rationalized **5k** might serve as a promising lead for a novel class of antitubulin agents for cancer treatments.

Microtubules, which formed by the self-assembly of *α*- and *β*-tubulin heterodimers, are an essential eukaryotic protein that plays critical roles in cell division, intracellular trafficking, cell migration and angiogenesis^1^,^2^,^3^,^4^. Therefore, anticancer therapy based on microtubule targeting agents (MTAs) is receiving growing attentions in drug discovery^5^. MTAs (Fig. 1) are known to interact with tubulin *via* at least four binding sites: the laulimalide, taxane (stabilisers of microtubules), vinca alkaloid and colchicine sites (destabilisers of microtubules)^6^,^7^. The taxanes and vinca alkaloids have been approved in clinical practice as antitumor agents^8^,^9^. Despite numerous efforts, none of the colchicine-binding site inhibitors (CBSIs) reached the market^10^, thus the discovery of antitubulin CBSIs is still of substantial efforts, which have led to the identification of several classes of inhibitors.

Combretastatin A-4 (CA-4), a natural *cis*-stilbene derivative isolated from the bark of South Africa tree *Combretum caffrum*, is a highly effective natural antitubulin agent strongly effecting microtubules dynamics by binding to the colchicine site which exhibited excellent antiproliferative activity against a wide panel of cell lines^11^,^12^. However, two major obstacles of CA-4 remain unsolved: the poor solubility and acquired multidrug resistance^13^, thus stimulating significant interest in diverse CA-4 analogs. Despite initial SARs study with CA-4 analogs indicated that the *cis*-olefin configuration at linking bridge was essential for optimal activity^14^, deficiency of the *cis*-olefin prone to isomerize to chemically more stable *trans*-configuration resulted in a dramatic loss in antitumor potency. Therefore, a variety of heteroaromatic-linkaged CA-4 derivatives, such as imidazole^15^, isoxazole^16^, thiophene^17^, pyrazole^18^ and indole group^19^ as link-bridge, were disclosed to retain the appropriate configuration of two adjacent aromatic A-ring and B-ring required for bioactivity. For example, the oxazoline derivative **a** (Fig. 1) with oxazoline motif and indolyl scaffold instead of vinyl linkage and B-ring in CA-4 respectively, displayed potent cytotoxic activity. Also, structurally related CA-4 analogue **b** that incorporated an indolyl moiety was reported as potent tubulin polymerization inhibitor^19^.

Crolibulin, a CBSIs under phase II clinical trials^20^,^21^, is active against various experimental tumors and exhibit potent inhibition of mitosis at the G2/M stage^22^, while its undue toxicity, for example, cardiovascular toxicity and neurotoxicity, impinged its clinical development^23^,^24^. The SARs study revealed that the cyano group and aromatic A-ring of Crolibulin are essential elements for exerting biological activity^25^,^26^,^27^. Thus, in an attempt to design novel antitubulin agents targeting colchicines-binding site, we were stimulated to optimize the chromene moiety by applying bioisosterism strategy.

Recently, the concept of “privileged medicinal scaffolds” has emerged as one of the guiding principles of drug discovery^28^,^29^,^30^. These frameworks commonly consist of a rigid heterocyclic ring system that assigns well-defined orientation of appended functionalities for target recognition. In this regard, imidazo[1,2-*a*]pyridines (IMPs) skeleton acted as the privileged medicinal scaffold are of particular utility serving for the generation of small-molecule ligands with pronounced antitumor, antiviral or anti-inflammatory efficacy^31^,^32^,^33^, such as GSK923295A^34^ and Alpidem^35^. Herein, our efforts were towards to optimize the 4*H*-chromenes-3-carbonitrile scaffold in Crolibulin by replacement with imidazo[1,2-*a*]pyridine-8-carbonitrile fragment based on bioisosterism strategy.

In the continuation of SARs study of restricted CA-4 analogs, we envisioned that imidazo[1,2-*a*]pyridine-8-carbonitrile fragment could serve as link-bridge instead of the *cis*-olefin in CA-4, while the favorable indolyl group was introduced into the B-ring by employing hybridization principle. Therefore, a series of novel 7-phenylimidazo[1,2-*a*]pyridine-8-carbonitrile derivatives conjugated with indolyl moiety (**5a**–**5v**) as antitubulin agents were designed and synthesized. Eventually, diverse substituents were introduced to the terminal phenyl fragment and indole portion attempting to investigate the influence on antitumor efficacy by regulating the electronic and steric effects. To further optimize properties of this series of compounds, various hydrophilic *N*-aliphatic aminomethyl groups were imported into IMPs moiety at C-2 position to afford derivatives **7a**–**7h.**

In this study, a series of novel 7-arylimidazo[1,2-*a*]pyridine-8-carbonitrile derivatives bearing indolyl moiety were firstly designed and optimized as colchicines-binding-site targeted antitubulin agents. All the compounds were prepared *via* a concise three-step process involving acylation reaction, one-pot coupling reaction and cyclization transformation, and their structures were determined by MS, ^1^H-NMR, ^13^C-NMR and element analysis. Finally, antiproliferative activities of the target compounds were evaluated against five human cancer cell lines and one normal human cell in cellular assay. Also, the primary mechanism of the lead compound **5k** on the inhibition of tubulin assembly was examined *in vitro* by cell cycle arrest, immunofluorescence assay and colchicine competition assay, which indicating a G2/M phase arrest and cellular microtubule depolymerization by binding to the colchicine site, as was in accordance with the docking mode study.

## Result and Discussion

### Chemistry

**Figure 2:**
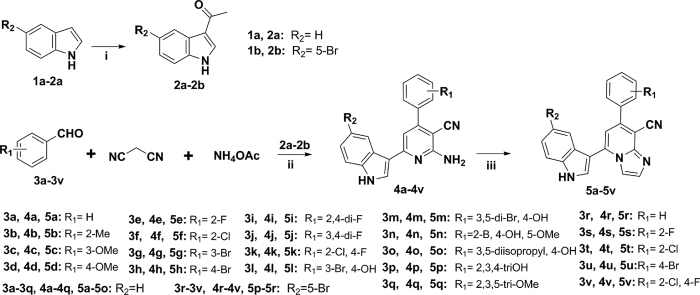
Reagents and conditions. (i) stannic chloride, acetyl chloride, dichloromethane, nitromethane, 0 °C to r.t., 3.5h; (ii) ammonium acetate, toluene, reflux; (iii) 2-chloroacetaldehyde, sodium bicarbonate, ethanol, reflux.

series of 5-indolyl-7-arylimidazo[1,2-*a*]pyridine-8-carbonitrile derivatives (**5a-5v**) and their intermediates were synthesized according to the pathways described in Fig. 2. Acylation reaction of 1*H*-indole or 5-bromo-1*H*-indole with acetyl chloride in the presence of stannic chloride in dichloromethane and nitromethane co-solvent for 3.5 h afforded compounds **2a**–**2b**. Then, 2-amino-4,6-diarylnicotinonitrile derivatives **4a**–**4v** could be concisely assembled in a one-pot coupling reaction of four components, namely **2a** or **2b**, aromatic formaldehyde, malononitrile and ammonium acetate. In this section, the one-pot coupling protocol as an efficient method for preparation of pyridines, has been developed allowing reaction conditions to be accessed in organic synthesis^36^. Subsequently, **4a**–**4v** were subjected to further cyclization transformation with 2-chloroacetaldehyde in refluxing ethanol on exposure to sodium bicarbonate gave rise to the desired target compounds **5a**–**5v** in good yields (Supplementary Information).

**Figure 3:**
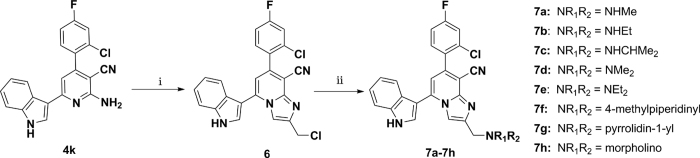
Reagents and conditions. (i) 1,3-dichloropropan-2-one, ethanol, reflux; (ii) *N*-aliphatic amines, ethanol, reflux.

The target compounds **7a**–**7h** were prepared according to the method as depicted in Fig. 3. The synthesized **4k** reacted with 1,3-dichloropropan-2-one in ethanol refluxed for 8 h to generate 7-(2-chloro-4-fluorophenyl)-2-chloromethyl-5-(1*H*-indol-3-yl)imidazo[1,2-*a*]pyridine-8-carbonitrile (**6**). Eventually, nucleophilic substitution of intermediate **6** with a variety of *N*-aliphatic amines afforded the corresponding compounds **7a**–**7h** in satisfied yield (Supplementary Information).

### Bioactivity

#### *In vitro* antiproliferative activity and SARs study

To evaluate the antiproliferative activity, all synthesized derivatives (**5a**–**5v** and **7a**–**7h**) were investigated for their activity *in vitro* against a panel of cancer cell lines including HT-29 (human colon cancer), H460 (human lung cancer), A549 (human lung carcinoma), MKN-45 (human gastric cancer) and SMMC-7721 (human liver cancer) by MTT assay. CA-4 and Crolibulin were served as positive control, and the results expressed as half-maximal inhibitory concentration (IC_50_). IC_50_ values are the concentrations that cause 50% inhibition of cancer cell growth (μM). Data represent the mean values ± standard deviation (SD) of independent experiments performed in triplicate.

As shown in Table 1, all the evaluated compounds (**5a**–**5v**) bearing imidazo[1,2-*a*]pyridines (IMPs) scaffold showed moderate to significant cytotoxic activity against HT-29, H460, A549 and MKN45 cell lines with potency in a dozen nanomolar range, but displayed at least 2-fold less sensitivity on SMMC-7721 cell as indicated by the IC_50_ values. Moreover, most compounds exhibited excellent cytotoxicity against HT-29, reflecting good selectivity for colon cancer. Significantly, five compounds (**5e**, **5f**, **5i**, **5j** and **5k**) demonstrated prominent cytotoxicity with IC_50_ values ranging from 0.02 to 26.15 μM, which was comparable or superior to Crolibulin and CA-4. Notably, the candidate **5k** displayed pronounced activity with IC_50_ values of 0.02 μM, 0.05 μM, 0.57 μM, 0.14 μM and 1.22 μM against HT-29, H460, A549, MKN-45 and SMMC-7721 cell lines respectively, which was about 0.60- to 26-fold more potent than Crolibulin, and almost twice over the average of CA-4.

The SARs based on IC_50_ values (Table 1) showed that different substitution patterns of the aromatic A-ring and B-ring attached on IMPs skeleton had a vital impact on the cytotoxicity. It is worth noting that introduction of bromine group at position C-5 of B-ring resulted in a 1.5- to 13.8-fold decreased in potency as compared with the relative hydrogenous compounds (**5a** *vs.* **5r**, **5e** *vs*. **5s**, **5f** *vs*. **5t,** and **5h** *vs*. **5u**), in good agreement with the low expand ability of the B-ring pocket at the colchicine site. Obviously, embedment of substituents into A-ring (**5b**–**5n**) led to a dramatic increase in cytotoxicity against MKN-45 and A549 cells relative to non-substituted phenyl analogue **5a**. Interestingly, the halogenated phenyl analogue (**5h,** IC_50_ = 0.12 μM) was about 2.5-fold more potent than **5a** and almost 5.8-fold more active than Crolibulin (IC_50_ = 0.52 μM) on HT-29 cell, indicating the presence of electron-withdrawing halogen group on A-ring would impart enhanced activity. By contrast, the electron-donating groups, with the exception of compound **5b** with methyl group, weakened the biological activity slightly, as might be the reason of compounds **5e** and **5f** with 2-F or 2-Cl groups exhibited at least 1.7-fold increased potency over **5a** (H), **5c** (3-OMe) and **5d** (4-OMe). Generally, in contrast with the mono-electron-withdrawing functions on phenyl portion, the introduction of double-electron-withdrawing groups showed evidently improved cytotoxicity against H460 and A549 cell lines as well as a slight increase for other three cancer cells. In particular, shifting the mono-halogen group (e.g. **5e**) to a double halogen substitutions at both C-2 and C-4 positions (e.g. **5k**) turned out to enhance the biological activity, speculating the latter might form stronger interactions with target protein through hydrogen bonds or polar contact concluded from docking study. For instance, the 2,4-difluorophenyl analog **5i** (IC_50_ = 0.04 μM) was about 29-fold more active in H460 than the 3-bromophenyl analog **5h** (IC_50_ = 1.16 μM). While, a dramatic loss in cytotoxic potency was observed for derivatives **5m**–**5q** with tri-substituents on phenyl ring against five cell lines. In addition, compounds bearing small substituents, such as **5p** with 2,3,4-tri-OH (HT-29, IC_50_ = 1.48 μM) and **5q** with 2,3,5-tri-OMe (HT-29, IC_50_ = 0.46 μM), displayed more potent cytotoxicity than **5o** possessing a bulky 3,5-di-isopropyl-4-OH group (HT-29, IC_50_ = 92.63 μM), suggesting the substituted group at this region was fairly sensitive to a substituent’s size.

To further optimize structural skeleton and explore the extending SARs, we prepared a series of derivatives **7a**–**7h** by introducing several *N*-aliphatic amino groups into the position C-2 on the IMP skeleton of the most active candidate **5k**. The pharmacological data of **7a**–**7h** was illustrated in Table 2. Though inferior to the relative lead **5k**, the *in vitro* cellular assay revealed that these compounds exhibited promising anticancer activity with IC_50_ values ranging from 0.03 to 42.57 μM against tested five cancer cells (Table 2). Especially, it is noteworthy to mention that **7b** with ethylamino moiety (HT-29, IC_50_ = 0.03 μM) displayed the optimal antiproliferative activity which was about 17-fold and 1.7-fold more active than the reference Crolibulin (HT-29, IC_50_ = 0.52 μM) and CA-4 (HT-29, IC_50_ = 0.05 μM), respectively. However, an increase in the steric hindrance or a replacement with tertiary amino group (e.g. dimethylamino, 4-methylpiperidinyl and morpholinyl, *et al*.) strongly diminished the efficiency as observed in compounds **7d**, **7f** and **7h.** The negative effect on cytotoxic activity of a bulk amino group might be attributed to prevent the test molecule from reaching the colchicine-binding-site of tubulin and thus result in a low activity. To our delight, compounds **7a**–**7h** exhibited pronounced activity against HT-29 cell superior to on other four cells with a 3- to 1000-fold increase in IC_50_ values, indicating the prominent selectivity on HT-29. Additional SARs and further optimization are in progress and will be reported in a due course.

The drug candidate potency depends not only on its cytotoxicity in malignant cells, but also on its relative lack of toxicity toward normal cells. Therefore, we evaluated the effects of compound **5b**, **5f** and **5k** on human fetal hepatocyte line L02 (non-cancer cell line) by MTT assay. As shown in Fig. 2B, three compounds almost had negligible effects on the normal cell, indicating a particularly effectivity and hypotoxicity of this series of derivatives.

#### 5k induces G2/M cell cycle arrest

**Figure 4:**
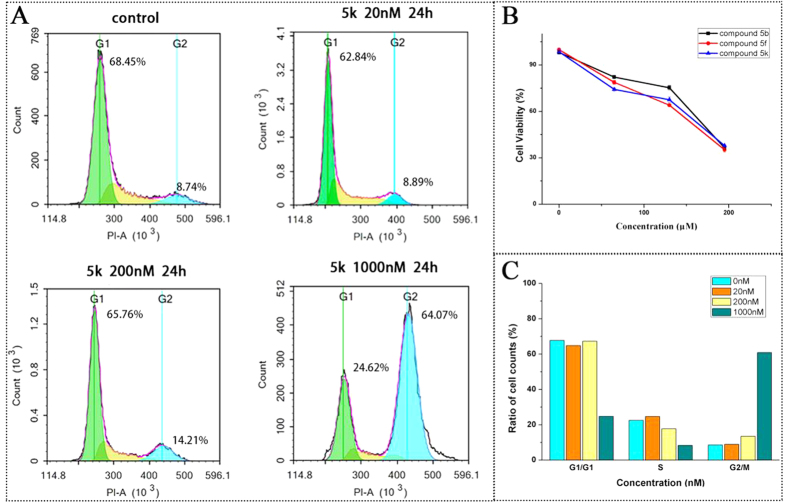
Effects of 5k on cell cycle progression in HT-29 cells. (**A**,**C)**: HT-29 cells were incubated with vehicle (DMSO, as CTL), 0, 20, 200 and 1000 nM of **5k** for 24 h and collected for cell cycle analysis. (**B**): the effect of compounds **5b**, **5f** and **5k** on human fetal hepatocyte line L02. Data are expressed as the mean ± SEM.

The structural similarities between the target compounds (**5a**–**5v** and **7a**–**7h**) with CA-4 and Crolibulin, as well as the promising cytotoxic activity led us to hypothesize that these compounds exert their antiproliferative properties through inhibition of tubulin dynamics, thus inducing mitotic arrest in cancer cells. To test the hypothesis, the lead compound **5k** was therefore subjected to human colon cancer HT-29 cell to examine for its effect on cell cycle progression and further to identify whether cell apoptosis was involved in the inhibition of HT-29 cell growth. HT-29 cells were incubated for 24 h with increasing concentrations of **5k** at concentration ranging from 20 nM to 1000 nM, and the percent of cells in each phase of the cell cycle was determined by flow cytometry (Fig. 4). Controls were treated with drug vehicle DMSO. Cell cycle analysis revealed that the cells accumulated in G2/M phase of the cell cycle in a concentration-dependent manner, whereas the control cells were mainly in the G1 phase. Results presented in Fig. 2A and C showed that treatment with **5k** (0 nM, 20 nM, 200 nM and 1000 nM) resulted in the percentage of cells in the G2/M phase increased from 8.89% to 64.07% significantly, indicating a pronounced cell cycle arrest in G2/M phase. And this effect is characteristic of antimitotic agents disrupting microtubule assembly.

#### Effect of 5k on microtubule depolymerization in immunofluorescence studies

**Figure 5:**
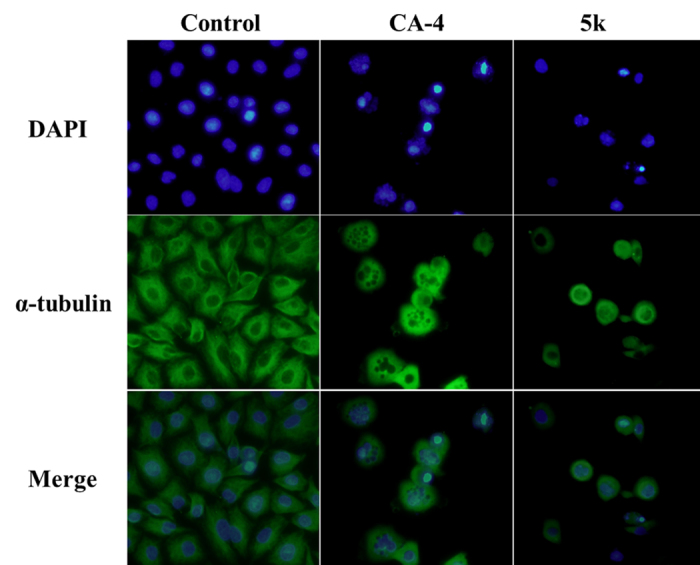
Effect of 5k on microtubules and nuclear condensation. **5k** induces microtubules depolymerization in HT-29 cells. HT-29 cells were treated with CA-4 (20 nM) or **5k** (32.5 nM) for 12 h. After incubation, cells were fixed, reacted with monoclonal anti-tubulin antibody at 4 °C overnight and then reacted with fluorescein isothiocyanate (FITC) conjugated secondary antibody, DAPI was used for nuclear counter-staining, the cellular microtubules were observed under fluorescence microscope (scale bar = 20 μm).

In order to further ascertain the characteristic cellular morphological changes that maybe relevant to the antitubulin activity of compound **5k**, we evaluated its effect on the microtubule network *via* tubulin immunostaining. The immunofluorescence analysis using the specific antibodies to *α*-tubulin revealed that the microtubule network of the cells in negative control group displayed intact organization and arrangement. **5k** (32.5 nM) and CA-4 (20 nM) treated cells exhibited cellular microtubule depolymerization with scattered short microtubule fragments in the cytoplasm of HT-29 cells (Fig. 5).

#### 5k binds to tubulin at the colchicine site

**Figure 6:**
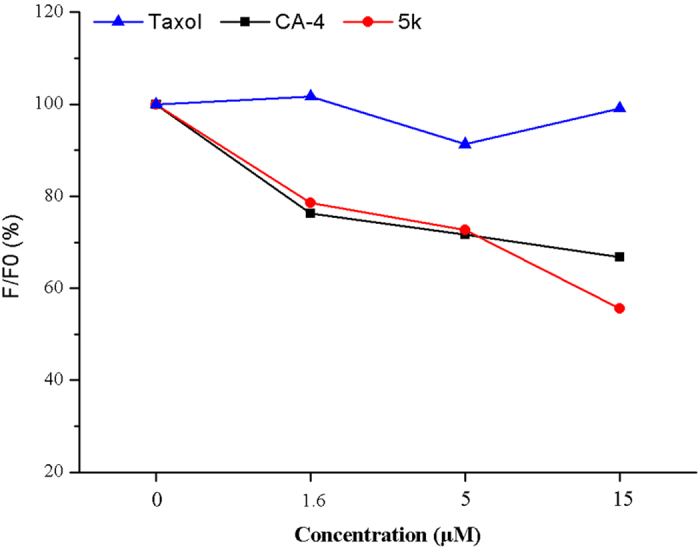
Colchicine competition assay. Tubulin was co-incubated with indicated concentrations of Taxol, CA-4 or **5k** for 1h, then 5 μM colchicine was added. The fluorescence was measured by Hitachi F-2500 spectrofluorometer.

To further elucidate the mechanism of 5-indolyl-7-arylimidazo[1,2-*a*]pyridine-8-carbonitrile derivatives interfering with microtubule formation, a competition binding assay was employed to examine whether the selected **5k** directly binds to the colchicine site. As reported, competition between the test compound and colchicine for the binding site will decrease the intrinsic fluorescence of colchicine-tubulin complex by reducing the amount of colchicine bound, which could be used as an index for **5k** competition with colchicine in tubulin binding^37^. As depicted in Fig. 6, 5k decreased the intrinsic colchicine fluorescence in a dose-dependent manner while Taxol exerts scarcely effect on the complex fluorescence for the reason that it binds at a different site on tubulin. Therefore, the results showed that compound **5k** strongly bound to the colchicine binding domain of tubulin compared with the known microtubule polymerization inhibitor CA-4. Together, all these findings showed that in both molecular and cellular levels, tubulin polymerization was disturbed by **5k** involving in the colchicine-binding-site, denoting the potential tubulin-targeting activity possessed by this compound.

#### Molecular modeling of 5k in the colchicine-binding site of tubulin

To make sense of the cytotoxic data observed and the most probable orientation of compound **5k** in the colchicine-binding site, molecular docking simulation of **5k** to tubulin was carried out using Discovery Studio Program 3.0. The X-ray crystal structure of the DAMA-colchicines-tubulin complex (PDB code: 4O2B)^38^ was used as the tubulin protein template. The 3D structure of **5k** was built using the Sybyl sketch module followed by energy minimization using the Triposforce field.

**Figure 7:**
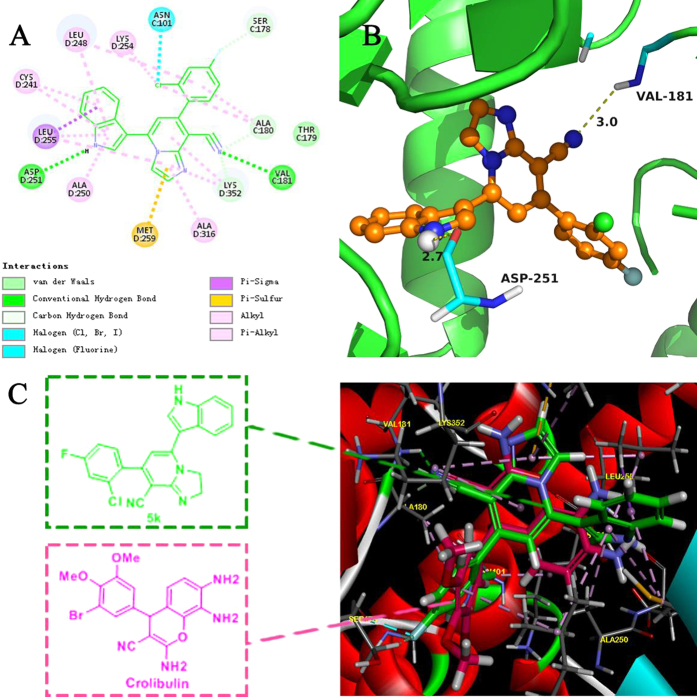
The binding mode between the active conformation of 5k and tubulin. (**A**) 2D diagram of the interaction between **5k** and the colchicines binding site. (**B**) 3D diagram of the interaction between **5k** and the colchicine binding site. For clarity, only interacting residues are displayed. The H-bond (yellow arrows) is displayed as dotted arrows. (**C**) Predicted modes for **5k** (green) and Crolibulin (pink) binding at the colchicine-binding site of tubulin (PDB code: 4O2B), and overlapping with each other.

As expected, docking studies showed that **5k** occupies the colchicine binding site shown in the Fig. 7B,C. The main binding driving force was thought to be shape matching with hydrophobic interaction between **5k** and tubulin (Fig. 7A). Also, **5k** formed two hydrogen bonds with Val181 of *α*-tubulin and Asp251 of *β*-tubulin shown in Fig. 7B. Concretely, the imidazo[1,2-*a*]pyridine (IMP) skeleton could link up to Met259, Ala316, Lys352 and Leu255 by hydrophobic interaction (Fig. 7A), suggesting that introduction of IMP moiety instead of chromenes scaffold retains tubulin-binding potency. The cyano group on the IMP framwork is engaged in a hydrogen bond with the amino group of Val181 of *a*-tubulin (Fig. 7B). And also, the indole group was involved in the hydrogen bonding with the Asp251 and hydrophobic interactions with the Leu248, Cys241, Leu255 and Ala250, which revealed that indole moiety existing in the structure of **5k** could foster potent binding affinity leading to significant antiproliferative activities (Fig. 7A). Moreover, the substituted phenyl moiety also can form hydrophobic interactions with the hydrophobic pocket made up by Ala180, Thr179, Ser178, Asn101, Lys254 and Leu248. Notably, the halogen at both C-2 and C-4 positions of the phenyl ring forms hydrophobic interactions well with the hydrophobic P1 pocket, as may be the reason that introducing halogen groups could enhance the antitubulin activity. Thus, the results from the docking study were in accordance with the SARs analysis (e.g. **5f, 5j** and **5k**).To further understand the detailed binding mode of the candidate **5k**, we thus superimposed Crolibulin and **5k** in a 3D model (Fig. 7C). The result disclosed **5k** was oriented in the same direction with Crolibulin at the colchicine site. Besides, the structural features of **5k** were also in accordance with the common pharmacophores of ligands of the colchicine-binding site reported in literature^39^. Therefore, the binding mode revealed the posture of **5k** is similar to Crolibulin binding at the colchicine site of tubulin.

## Conclusion

In this paper, in an attempt to develop promising tubulin polymerization inhibitors targeting colchicine-binding site, a series of 5-indolyl-7-arylimidazo[1,2-*a*]pyridine-8-carbonitrile derivatives (**5a**–**5v** and **7a**–**7h**) were discovered according to bioisosterism and hybridization principles. This novel class of compounds was efficiently synthesized *via* a concise three-step reaction involving acylation reaction, one-pot coupling reaction and cyclization transformation, and the resultant thirty title compounds were determined by MS, ^1^H-NMR, ^13^C-NMR and element analysis. Evaluation of target compounds against a panel of cancer cell lines (HT-29, A549, MKN-45, MDA-MB-231 and SMMC-7721) resulted in the discovery of five promising compounds (**5e**, **5f**, **5i**, **5j** and **5k**) with the IC_50_ value in a dozen nanomolar range, indicating a great potency as antitubulin agents. Exploration of SARs based on IC_50_ values led to the identification of **5k** as a prominent lead, which possessed remarkable antitumor potency against all the tested cancer cells with IC_50_ values ranging from 0.02 μM to 1.22 μM superior to references CA-4 and Crolibulin. In contrast, its cytotoxic effect on normal human fetal hepatocyte cell line (L02) was minimal. Importantly, the cell cycle analysis, immunofluorescence assay and the colchicine competition assay confirmed that candidate **5k** could inhibit cellular tubulin polymerization by binding to the colchicine site, interfere with the mitosis, and at the end lead to G2/M cell cycle arrest *in vitro*. Moreover, a molecular docking mode revealed that **5k** could not only form critical hydrogen bonding interactions and hydrophobic interactions with tubullin but also overlap well with Crolibulin in 3D model, which is consistent with the observed SARs for this class of compounds.

In summary, this is the first report of a novel series of 5-indolyl-7-arylimidazo[1,2-*a*]pyridine-8-carbonitrile derivatives as potent tubulin-targeting agents and thus, describe the SARs-guided discovery process that lead to a candidate **5k** as a potential antitubulin agent targeting colchicines-binding-site. All of these finding suggest the molecular basis of **5k** to serve as a probe in the characterization of a new class of tubulin polymerization inhibitors.

## Experimental Procedures

### Reagents and instrumentation

All melting points were obtained on a Büchi Melting Point B-540 apparatus (BüchiLabortechnik, Flawil, Switzerland) and were uncorrected. Mass spectra (MS) were taken in ESI mode on Agilent 1100 LC-MS (Agilent, palo Alto, CA, USA). ^1^H NMR and ^13^C NMR spectra were performed using Bruker 400 MHz and 100 MHz spectrometers (Bruker Bioscience, respectively, Billerica, MA, USA) with TMS as an internal standard. Column chromatography was run on silica gel (200–300 mesh) from Qingdao Ocean Chemicals (Qingdao, Shandong, China). Unless otherwise noted, all materials were obtained from commercially available sources and were used without further purification.

### Chemistry section

#### General procedure for Preparation of compounds (2a–2b)

A 500 mL oven-dried three-necked flask was deal with septa and drained/backfilled with nitrogen gas (N_2_) three times before starting the reaction. A solution of anhydrous stannic chloride (62.4 g, 240 mmol) in dichloromethane (150 mL) was added to a solution of 1*H*-indole or 5-bromo-1*H*-indole (200 mmol) in dry dichloromethane (200 mL) at −5 °C and the mixture was stirred at 0 °C for 30 min. Then a solution of acetyl chloride (15.6 g, 200 mmol) in dry dichloromethane/nitromethane co-solvent (1:1, 50 mL) was added successively. Subsequently, the resulting mixture was stirred at 0 °C for 30 min and then warmed to room temperature stirring for 3 h. After monitored by TLC, the reaction mixture was quenched with ice-water, the suspension solution was filtered and the filtrate was extracted with ethyl acetate (100 mL × 2). Afterward, the combined organic phase was washed with water and brine, dried over Na_2_SO_4_, and concentrated in *vacuo*. The removal of the solvent yielded a brown residue that was purified by washing-up with ethanol for 1 h to furnish **2a** as a white solid.

#### General procedure for the preparation of compounds (4a–4r)

To a solution of substituted benzaldehydes (100 mmol) in toluene (250 mL) was added **2a** or **2b** (100 mmol), ammonium acetate (18.48 g, 240 mmol) and malononitrile (6.6 g, 100 mmol) at room temperature. Then the solution was refluxed until the completion of the reaction indicated by TLC (about 8 h). Up on cooling to room temperature, the solvent was removed under vacumm, and then, the residue was triturated with ethanol (100 mL) and filtered to give the corresponding crude product. Afterward, the crude product was transited to anhydrous ethanol (120 mL) and stirred overnight at room temperature. Finally, the suspension liquid was filtered off, dried over MgSO_4_ to afford **4a**-**4v.**

#### General procedure for the preparation of compounds (5a - 5 v)

To a solution of **4a-4v** (1.6 mmol) and sodium bicarbonate (0.14 g, 1.6 mmol) in absolute ethanol (30 mL) were added to 40% 2-chloroacetaldehyde (3.14 g, 16 mmol). The mixture was refluxed and stirred for 4 h. After the solvent was evaporated, the residue was obtained by filtration and washed with distilled water (30 mL) to give the corresponding pure compounds **5a-5v**.

#### Preparation of 7-(2-chloro-4-fluorophenyl)-2-chloromethyl-5-(1H-indol-3-yl)imidazo[1,2-a] pyridine-8-carbonitrile (6)

To a mixture of **4k** (5.0 g, 13.8 mmol) and 1,3-dichloropropan-2-one (18.2 g, 144.1 mmol) in ethanol (50 mL) were added and refluxed for 10 h. After the completion of thereaction indicated by TLC, the solvent was evaporated and then the residue was washed with ethyl ether (50 mL) for 2 h to give the corresponding crude product in 64.7% yield.

#### General procedure for the preparation of compounds (7a–7h)

A stirring mixture of **6** (0.5 g, 1.2 mmol) and an appropriate *N*-aliphatic amines (1.8 mmol) in ethanol (15 mL) was stirred at reflux for 5 h. After cooling to room temperature, the solvent was evaporated in *vacuum* to afford yellow residue. The formed precipitate was dissolved in DMF and purified on a silica gel column (petroleum ether/ethyl acetate, 3:1) to give pure target product **7a**–**7h**.

(Thirty target compounds were determined by MS, ^1^H-NMR, ^13^C-NMR and element analysis, and the detailed information is in Supplementary Information).

### Biological section

Cell lines and culture conditions.The human colon carcinoma cell line HT-29, the human pulmonary carcinoma cell line A549, the human gastric carcinoma cell line MKN-45, the human breast cancer cell line MDA-MB-231 and human heptocarcinoma cellline SMMC-7721were cultured in RPMI-1640 mediumcontaining 10% FBS, 100 U/mL streptomycin and 100 U/mL penicillinat 37 °C in humidified atmosphere with 5% CO_2_. All of the cells were purchased from the American Type Culture Collection (ATCC, Manassas, VA).

#### MTT assay

The antiproliferative activities *in vitro* of Crolibulin and all the target compounds were determined by an MTT assay^40^. Briefly, cells were seeded into 96-well plates at a density of 1–3 × 10^4^/well (depends on the cell growth rate). 24 h later, triplicate wells were treated with media and the agents. After 72 h ofincubation at 37 °C in 5% CO_2_, the drug containing medium wasremoved and replaced by 100 mL of fresh medium with 5 mg/mLMTT solution. After 4 h of incubation, the medium with MTT was removed, and 100 mL of DMSO was added to each well. The plates were gently agitated until the purple formazan crystals were dissolved, and the OD_490_ was determinedusing a microplate reader (MK3, Thermo, Germany). The data were calculated and plotted as the percent viability compared to the control. The 50% inhibitory concentration (IC_50_) was defined as theconcentration that reduced the absorbance of the untreated wells by 50% of the vehicle in the MTT assay.

#### Cell cycle phase distribution

For flow cytometric analysis of DNA content, cells inexponential growth were treated with compound for 24 h^41^. The cells treated with compoundwere collected, washed twice with PBS, and then fixed with 75% alcohol overnight. Then, the cells were washed with PBS and resuspended in 100 μL of PBS, 200 mg/mL RNase wasadded for 30 min to eliminate the interference of RNA, and 20 mL/L propidium iodide (PI; Sigma) was added for 30 min.Then, the cells were washed, and the DNA content wasdetected by flow cytometer (BD Accuri C6).

#### Immunofluorescence staining assay

Immunostaining was carried out to detect microtubule associatedtubulin protein after exposure to CA-4 and **5k**^42^. The HT-29 cells were seeded at 1 × 10^4^ per well on a 24 wellplate and grown for 24 h. Cells were treated with CA-4 or **5k** for 12 h. Cells in the control group were treated with culture medium. The control and treated cells were fixed with 4% formaldehydein PBS for 30 min at −20 °C, then washed twice with PBS and permeabilized with 0.1% (v/v) Triton X-100 in PBS for 5 min. Then, the cells were blocked with 3% bovine serum albumin(BSA) in PBS for 30 min. The primary *α*-tubulin antibody wasdiluted (1:100) with 2% BSA in PBS and incubated overnight at 4 °C. The cells were washed with PBS to remove unbound primaryantibody and then cells were incubated with FITC-conjugated antimousesecondary antibody, diluted (1:100) with 2% BSA in PBS, for 2 h at 37 °C. The cells were washed with PBS to remove unbound secondary antibody, nucleus was stained with 4,6-diamino-2-phenolindoldihydrochloride (DAPI) and then, immunofluorescencewas detected using a fluorescence microscope (Olympus,Tokyo, Japan).

#### Competitive tubulin-binding assay

Tubulin was co-incubated with various concentrations of Taxol, CA-4 or **5k** respectively at 37 °C for 1 h. Then colchicine was added to a final concentration of 5 μM^43^. Fluorescence was determined using a Hitachi F-2500 spectrofluorometer (Tokyo, Japan) at excitation wavelengths of 365 nm and emission wavelengths of 435 nm. Blank values (buffer alone) as background were subtracted from all samples. Then the inhibition rate (IR) was calculated as follows: IR = (F ÷ F0) × 100% where F0 is the fluorescence of the 5 μM colchicine-tubulin complex, and F is the fluorescence of a given concentration of Taxol, **5k** or CA-4 (0 μM, 1.6 μM, 5 μM and 15 μM) competition with the 5 μM colchicine-tubulin complex. Taxol, not binding in the colchicine-site of tubulin, was added as a negative control.

#### Molecular Docking

The molecular modelling studies were performed with Accelrys Discovery Studio 3.0^44^. The crystal structure of tubulin complexedwith DAMA-colchicine (PDB code: 4O2B) was retrieved from the RCSB Protein Data Bank (http://www.rcsb.org/pdb/). In the docking process, the protein protocol was prepared by several operations, including standardization of atom names and insertion of missingatoms in residues. Then, the receptor model was typed with the CHARMm force field and a binding sphere with radius of 15.0 Å was defined through the original ligand (DAMA-colchicine) as the binding site. The Crolibulin and **5k** were drawn with Chemdraw and fully minimized using the CHARMm force field. Finally, they were docked into the binding site using the CDOCKER protocol with the default settings. And figures were prepared using PyMOL (The PyMOL Molecular Graphics System, Version 1.4.1. Schrödinger, LLC).

### Statistical analysis

Statistical analysis was performed with Origin Version 8.0 software package. Data were expressed as means ± standard deviation (SD). Comparisons between groups were performed with analysis of non-parametric test.

## Additional Information

**How to cite this article**: Zhai, X. *et al*. Discovery and Optimization of Novel 5-Indolyl-7-arylimidazo[1,2-*a*]pyridine-8-carbonitrile Derivatives as Potent Antitubulin Agents Targeting Colchicine-binding Site. *Sci. Rep.* **7**, 43398; doi: 10.1038/srep43398 (2017).

**Publisher's note:** Springer Nature remains neutral with regard to jurisdictional claims in published maps and institutional affiliations.

## Supplementary Material

Supplementary Information

## Figures and Tables

**Table 1:** Structures and cytotoxicity of compounds 5a–5v.

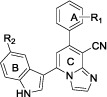							
Compd.	R_1_	R_2_	IC_50_ (μM) ±SD^a^				
			HT29	H460	A549	MKN45	SMMC-7721
**5a**	H	H	0.30 ± 0.04	2.79 ± 0.52	86.42 ± 7.33	79.67 ± 8.10	89.43 ± 2.60
**5b**	2-Me	H	0.16 ± 0.04	46.28 ± 5.26	0.23 ± 0.04	1.52 ± 0.15	0.82 ± 0.06
**5c**	3-OMe	H	0.24 ± 0.01	17.50 ± 1.53	12.23 ± 1.89	1.24 ± 0.08	76.15 ± 8.98
**5d**	4-OMe	H	0.43 ± 0.03	9.61 ± 0.76	15.47 ± 2.13	1.64 ± 0.07	71.53 ± 9.43
**5e**	2-F	H	0.09 ± 0.01	1.16 ± 0.12	5.67 ± 0.23	0.96 ± 0.06	26.15 ± 2.82
**5f**	2-Cl	H	0.07 ± 0.004	0.18 ± 0.08	0.95 ± 0.07	0.38 ± 0.06	13.83 ± 2.23
**5g**	3-Br	H	>100	6.89 ± 0.67	>100	64.0 ± 7.14	79.20 ± 6.87
**5h**	4-Br	H	0.12 ± 0.02	0.95 ± 0.03	8.54 ± 0.93	2.76 ± 0.47	53.45 ± 7.76
**5i**	2,4-di-F	H	0.08 ± 0.001	0.04 ± 0.001	0.12 ± 0.03	0.03 ± 0.006	13.56 ± 2.38
**5j**	3,4-di-F	H	0.03 ± 0.009	0.076 ± 0.01	0. 46 ± 0.06	1.43 ± 0.12	18.54 ± 2.76
**5k**	2-Cl, 4-F	H	0.02 ± 0.001	0.05 ± 0.008	0.57 ± 0.08	0.14 ± 0.03	1.22 ± 0.20
**5l**	3-Br, 4-OH	H	0.92 ± 0.08	1.07 ± 0.12	>100	10.72 ± 1.85	80.15 ± 7.54
**5m**	3,5-di-Br, 4-OH	H	2.13 ± 0.12	1.15 ± 0.13	2.45 ± 0.37	18.32 ± 2.13	20.47 ± 3.53
**5n**	2-Br, 4-OH, 5-OMe	H	1.16 ± 0.13	9.61 ± 0.74	80.16 ± 9.68	14.54 ± 2.43	97.16 ± 6.54
**5o**	3,5-di-isopropyl, 4-OH	H	92.63 ± 5.38	42.56 ± 5.17	96.16 ± 7.62	>100	77.64 ± 6.43
**5p**	2,3,4-tri-OH	H	1.48 ± 0.27	0.25 ± 0.031	1.18 ± 0.21	9.67 ± 0.73	10.44 ± 1.64
**5q**	2,3,5-tri-OMe	H	0.46 ± 0.03	0. 64 ± 0.04	1.75 ± 0.13	1.74 ± 0.15	27.36 ± 2.32
**5r**	H	Br	1.74 ± 0.14	11.43 ± 1.68	67.32 ± 7.43	7.38 ± 0.63	>100
**5s**	2-F	Br	2.86 ± 0.38	17.25 ± 1.34	67.32 ± 4.59	31.25 ± 5.79	>100
**5t**	2-Cl	Br	0.58 ± 0.06	80.71 ± 6.98	>100	56.82 ± 4.29	>100
**5u**	4-Br	Br	0.73 ± 0.09	1.93 ± 0.33	21.39 ± 2.58	38.14 ± 4.42	79.32 ± 5.14
**5v**	2-Cl, 4-F	Br	0.26 ± 0.03	2.13 ± 0.24	3.57 ± 0.23	7.35 ± 0.54	56.34 ± 4.87
**CA-4**			0.05 ± 0.01	0.08 ± 0.01	0.43 ± 0.053	0.11 ± 0.02	1.92 ± 0.11
**Crolibulin**			0.52 ± 0.02	0.03 ± 0.007	ND	0.17 ± 0.03	ND

^a^SD: standard deviation.

**Table 2:** Structures and cytotoxicity of compounds 7a–7 h.

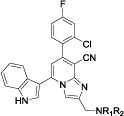						
Compd.	-NR_1_R_2_	IC_50_ (μM) ± SD^a^				
		HT29	H460	A549	MKN45	SMMC-772
**7a**	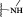	0.04 ± 0.001	1.29 ± 0.21	5.34 ± 0.67	12.19 ± 1.89	26.98 ± 3.06
**7b**	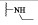	0.03 ± 0.003	0.96 ± 0.06	3.54 ± 0.41	20.47 ± 2.32	21.56 ± 3.16
**7c**	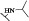	0.33 ± 0.04	0.96 ± 0.07	6.18 ± 0.71	22.35 ± 2.51	36.67 ± 4.92
**7d**	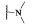	0.19 ± 0.02	0.71 ± 0.05	7.53 ± 0.63	16.56 ± 1.13	22.13 ± 3.41
**7e**	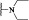	0.21 ± 0.03	0.67 ± 0.05	4.87 ± 0.38	13.75 ± 1.79	24.61 ± 2.73
**7f**	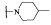	0.56 ± 0.07	0.85 ± 0.06	20.28 ± 2.32	1.63 ± 0.28	11.35 ± 2.15
**7g**	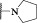	0.19 ± 0.02	1.32 ± 0.12	8.79 ± 0.62	19.17 ± 1.35	42.57 ± 4.12
**7h**	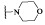	0.92 ± 0.14	1.27 ± 0.14	16.86 ± 1.98	36.31 ± 5.15	28.68 ± 1.79
**CA-4**		0.05 ± 0.01	0.08 ± 0.01	0.43 ± 0.053	0.11 ± 0.02	1.92 ± 0.11
**Crolibulin**		0.52 ± 0.02	0.03 ± 0.007	ND	0.17 ± 0.03	ND

^a^SD: standard deviation.

**Figure 1:**
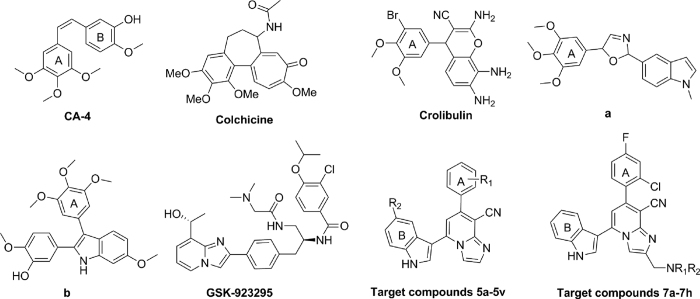
The representative antitubulin agents and target compounds (5a–5v and 7a–7h).
