# Supplementary material for: Discovery and Optimization of Novel 5-Indolyl-7-arylimidazo[1,2-a]pyridine-8-carbonitrile Derivatives as Potent Antitubulin Agents Targeting Colchicine-binding Site
Source: Sci Rep. 2017 Feb 27;7:43398. doi: 10.1038/srep43398 (PMC5327470; doi:10.1038/srep43398)
Supplement: Supplementary Information [file srep43398-s1.doc]

**Discovery and Optimization of Novel 5-Indolyl-7-arylimidazo[1,2-*a*]pyridine-8-carbonitrile Derivatives as Potent Antitubulin Agents Targeting Colchicine-binding Site**

Xin Zhai^1^, Xiaoqiang Wang^1^, Jiao Wang^1^, Jin Liu^1^, DaiyingZuo^2^, Nan Jiang^1^, Tianfang Zeng^1^, Xiuxiu Yang^1^, Tongfei Jing^1^ & Ping Gong^1^

^1^Key Laboratory of Structure-Based Drug Design and Discovery, Ministry of Education, Shenyang PharmaceuticalUniversity, 103 Wenhua Road, Shenhe District, Shenyang, 110016, China. ^2^Department of Pharmacology, Shenyang Pharmaceutical University, 103 Wenhua Road, Shenhe District, Shenyang, 110016, China. Correspondence and requests for materials should be addressed to X.Z. (email: zhaixin_syphu@126.com) or P.G. (email: gongpinggp@126.com).

**Chemistry section.**

*5.1.1. Preparation of 1-(1H-indol-3-yl)ethan-1-one (****2a****).*

A 500mL oven-dried three-necked flask was deal with septa and drained/backfilled with nitrogen gas (N_2_) three times before starting the reaction. A solution of anhydrous stannic chloride (62.4 g, 240 mmol) in dichloromethane (150 mL) was added to a solution of 1*H*-indole (23.4 g, 200 mmol) in dry dichloromethane (200 mL) at -5 ºC and the reaction mixture was stirred at 0 °C for 30 min. Then a solution of acetyl chloride (15.6 g, 200 mmol) in dry dichloromethane / nitromethane co-solvent (1:1, 50 mL) was added successively. Subsequently, the resulting mixture was stirred at 0 °C for 30 min and then warmed to room temperature stirring for 3 h. After monitored by TLC, the reaction mixture was quenched with ice-water, the suspension solution was filtered and the filtrate was extracted with ethyl acetate (100 mL×2). Afterward, the combined organic phase was washed with water and brine, dried over Na_2_SO_4_, and concentrated in *vacuo*. The removal of the solvent yielded a brown residue that was purified by washing-up with ethanol for 1h to furnish **2a** as a white solid in 79.3% yield. MS (ESI) *m/z*: 160.1[M+H]^+^.

*5.1.2. 1-(5-bromo-1H-indol-3-yl)ethan-1-one (****2b****).*

Prepared from 5-bromo-1*H*-indole (200 mmol) according to the procedure of **2a** gave rise to **2b** as pale powder in 85.0 % yield. MS (ESI) *m/z*: 238.0 [M+H]^+^.

*5.2.General procedure for the preparation of compounds (****4a - 4r****）.*

To a solution of substituted benzaldehydes (100 mmol) in toluene (250 mL) was added **2a** or **2b** (100 mmol), ammonium acetate (18.48 g, 240 mmol) and malononitrile (6.6 g, 100 mmol) at room temperature. Then the solution was refluxed until the completion of the reaction indicated by TLC (about 8 h). Up on cooling to room temperature, the solvent was removed under vacumm, and then, the residue was triturated with ethanol (100 mL) and filtered to give the corresponding crude product. Afterward, the crude product was transited to anhydrous ethanol (120mL) and stirred overnight at room temperature. Finally, the suspension liquid was filtered off, dried over MgSO_4_ to afford **4a**-**4v.**

5.2.1. 2-amino-6-(1*H*-indol-3-yl)-4-phenylnicotinonitrile (**4a**).

Yield: 63.2 %; MS (ESI) *m/z*: 311.1 [M+H]^+^.

5.2.2. 2-amino-6-(1*H*-indol-3-yl)-4-(o-tolyl)nicotinonitrile (**4b**).

Yield:57.1%；MS (ESI) *m/z*: 325.1 [M+H]^+^.

5.2.3. 2-amino-6-(1*H*-indol-3-yl)-4-(3-methoxyphenyl)nicotinonitrile (**4c**).

Yield: 61.7%；MS (ESI) *m/z*: 341.2 [M+H]^+^.

5.2.4. 2-amino-6-(1*H*-indol-3-yl)-4-(4-methoxyphenyl)nicotinonitrile (**4d**).

Yield: 68.5%；MS (ESI) *m/z*: 341.1 [M+H]^+^.

5.2.5. 2-amino-4-(2-fluorophenyl)-6-(1*H*-indol-3-yl)nicotinonitrile (**4e**).

Yield: 75.84%；MS (ESI) *m/z*: 329.2 [M+H]^+^.

5.2.5. 2-amino-4-(2-chlorophenyl)-6-(1*H*-indol-3-yl)nicotinonitrile (**4f**).

Yield: 58.3%；MS (ESI) *m/z*: 345.1 [M+H]^+^.

5.2.5. 2-amino-4-(3-bromophenyl)-6-(1*H*-indol-3-yl)nicotinonitrile (**4g**).

Yield: 62.8%；MS (ESI) *m/z*: 389.0 [M+H]^+^.

5.2.6. 2-amino-4-(4-bromophenyl)-6-(1*H*-indol-3-yl)nicotinonitrile (**4h**).

Yield: 81.7%；MS (ESI) *m/z*: 389.0 [M+H]^+^.

5.2.7. 2-amino-4-(2,4-difluorophenyl)-6-(1*H*-indol-3-yl)nicotinonitrile (**4i**).

Yield: 59.7%；MS (ESI) *m/z*: 347.1 [M+H]^+^.

5.2.8. 2-amino-4-(3,4-difluorophenyl)-6-(1*H*-indol-3-yl)nicotinonitrile (**4j**).

Yield:69.4%；MS (ESI) *m/z*: 347.1 [M+H]^+^.

5.2.9. 2-amino-4-(2-chloro-4-fluorophenyl)-6-(1*H*-indol-3-yl)nicotinonitrile (**4k**).

Yield: 74.6%；MS (ESI) *m/z*: 363.0 [M+H]+.

5.2.10. 2-amino-4-(3-bromo-4-hydroxyphenyl)-6-(1*H*-indol-3-yl)nicotinonitrile (**4l**).

Yield: 74.3%；MS (ESI) *m/z*: 405.0 [M+H]^+^.

5.2.11.2-amino-4-(3,5-dibromo-4-hydroxyphenyl)-6-(1*H*-indol-3-yl)nicotinonitrile (**4m**).

Yield: 59.5%；MS(ESI) *m/z*: 482.9 [M+H]^+^.

5.2.12.2-amino-4-(2-bromo-4-hydroxy-5-methoxyphenyl)-6-(1*H*-indol-3-yl)nicotinonitrile (**4n**)

Yield: 64.4%，MS (ESI) *m/z*: 435.0 [M+H]^+^.

5.2.11.2-amino-4-(4-hydroxy-3,5-diisobutylphenyl)-6-(1*H*-indol-3-yl)nicotinonitrile (**4o**).

Yield: 67.8%；MS(ESI) *m/z*: 439.2 [M+H]^+^.

5.2.13.2-amino-6-(1*H*-indol-3-yl)-4-(2,3,4-trihydroxyphenyl)nicotinonitrile (**4p**).

Yield: 77.3%；MS (ESI) *m/z*: 459.2 [M+H]^+^.

5.2.14. 2-amino-6-(1*H*-indol-3-yl)-4-(2,3,5-trimethoxyphenyl)nicotinonitrile(**4q**).

Yield: 53.7%；MS (ESI) *m/z*: 401.2 [M+H]^+^.

5.2.15. 2-amino-6-(5-bromo-1*H*-indol-3-yl)-4-phenylnicotinonitrile(**4r**).

Yield: 64.2%；MS(ESI) *m/z*: 389.0 [M+H]^+^.

5.2.16. 2-amino-6-(5-bromo-1*H*-indol-3-yl)-4-(2-fluorophenyl)nicotinonitrile(**4s**).

Yield: 85.4%；MS(ESI) *m/z*: 407.0 [M+H]^+^.

5.2.17.2-amino-6-(5-bromo-1*H*-indol-3-yl)-4-(2-chlorophenyl)nicotinonitrile (**4t**).

Yield: 60.2%；MS (ESI) *m/z*: 422.9 [M+H]^+^.

5.2.18.2-amino-6-(5-bromo-1*H*-indol-3-yl)-4-(4-bromophenyl)nicotinonitrile (**4u**).

Yield: 66.2%；MS(ESI) *m/z*: 467.4 [M+H]^+^.

5.2.19.2-amino-6-(5-bromo-1*H*-indol-3-yl)-4-(2-chloro-4-fluorophenyl)nicotinonitrile (**4v**).

Yield: 69.2%；MS(ESI) *m/z*: 441.4 [M+H]^+^.

*5.3.* *General procedure for the preparation of compounds (****5a - 5v****).*

To a solution of **4a-4v** (1.6 mmol) and sodium bicarbonate (0.14 g，1.6 mmol) in absolute ethanol (30 mL) were added to 40% 2-chloroacetaldehyde (3.14 g, 16 mmol). The mixture was refluxed and stirred for 4 h. After the solvent was evaporated, the residue was obtained by filtration and washed with distilled water (30mL) to give the corresponding pure compounds **5a-5v**.

5.3.1. 5-(1*H*-indol-3-yl)-7-phenylimidazo[1,2-*a*]pyridine-8-carbonitrile (**5a**).

Yield: 86.8 % ; m.p.: 338.3-339.7 ºC; MS (ESI) m/z: 335.1 [M+H]^+^; ^1^H NMR (400 MHz, DMSO-*d*_6_) δ (ppm) 12.31 (s, 1H), 8.27 (d, *J* = 1.5 Hz, 1H), 8.13 (s, 1H), 7.82 (d, 3H), 7.62-7.55 (m, 5H), 7.29-7.25 (m, 2H), 7.18 (t, *J* = 7.6 Hz, 1H); Anal. calcd. for C_22_H_14_N_4_ (%): C, 79.02; H, 4.22; N, 16.76; Found (%): C, 78.98; H, 4.21; N, 16.81.

5.3.2. 5-(1*H*-indol-3-yl)-7-(o-tolyl)imidazo[1,2-*a*]pyridine-8-carbonitrile (**5b**).

Yield: 77.0 %; m.p.: 334.3-335.1 ºC; MS (ESI) m/z: 347.2 [M-H]^-^; ^1^H NMR (400 MHz, DMSO-*d*_6_) δ (ppm) 12.14 (s, 1H), 8.50 (d, *J* = 2.4 Hz, 1H), 8.07 - 7.99 (m, 2H), 7.77 (s, 1H), 7.65 - 7.59 (m, 1H), 7.57 - 7.51 (m, 1H), 7.50 - 7.45 (m, 1H), 7.42 - 7.36 (m, 2H), 7.36 - 7.29 (m, 3H), 2.28 (s, 3H); Anal. calcd. for C_23_H_16_N_4_ (%): C, 79.29; H, 4.63; N, 16.08; Found (%): C, 79.31; H, 4.59; N, 16.10.

5.3.3. 5-(1*H*-indol-3-yl)-7-(3-methoxyphenyl)imidazo[1,2-*a*]pyridine-8-carbonitrile (**5c**).

Yield: 83.3 %; m.p.: 316.5-318.7 ºC; MS (ESI) m/z: 365.1 [M+H]^+^; ^1^H-NMR (400 MHz, DMSO-*d*_6_) δ (ppm): 12.14 (s, 1H), 8.27 (d, *J* = 2.8 Hz, 1H), 8.12 (d, *J* = 1.2 Hz, 1H), 7.82 (d, *J* = 1.1 Hz, 1H), 7.60 (d, *J* = 5.6 Hz, 2H), 7.51 (t, *J* = 8.2 Hz, 1H), 7.41 - 7.33 (m, 2H), 7.31 - 7.23 (m, 2H), 7.19 (t, *J* = 7.8 Hz, 1H), 7.12 (dd, *J* = 8.2, 1.5 Hz, 1H), 3.85 (s, 3H); Anal. calcd. for C_23_H_16_N_4_O (%): C, 75.81; H, 4.43; N, 15.38; Found (%): C, 75.83; H, 4.41; N, 15.35.

5.3.4. 5-(1*H*-indol-3-yl)-7-(4-methoxyphenyl)imidazo[1,2-*a*]pyridine-8-carbonitrile (**5d**).

Yield:79.1 %; m.p.: 294.7-295.5 ºC; MS (ESI) *m/z*: 365.1 [M+H]^+^; ^1^H NMR (400 MHz, DMSO*-d_6_*) δ (ppm) 12.13 (s, 1H), 8.26 (d, *J* = 2.8 Hz, 1H), 8.12 (d, *J* = 1.2 Hz, 1H), 7.80 (d, *J* = 1.1 Hz, 1H), 7.71 (d, *J* = 8.1 Hz, 2H), 7.59 (t, *J* = 7.2 Hz, 2H), 7.40 (d, *J* = 8.0 Hz, 2H), 7.31 - 7.14 (m, 3H), 2.41 (s, 3H); Anal. calcd. for C_23_H_16_N_4_O (%): C, 75.81; H, 4.43; N, 15.38; Found (%): C, 75.83; H, 4.41; N, 15.35.

5.3.5.7-(2-fluorophenyl)-5-(1*H*-indol-3-yl)imidazo[1,2-*a*]pyridine-8-carbonitrile (**5e**).

Yield: 85.2 %; m.p.: 304.6-308.8 ºC; MS (ESI) *m/z*: 353.0 [M+H]^+^; ^1^H NMR (400 MHz, DMSO*-d_6_*) δ (ppm) 12.18 (s, 1H), 8.29 (d, J=2.7, 1H), 8.23 (d, J=0.9, 1H), 7.87 (d, J=0.7, 1H), 7.82 - 7.73 (m, 1H), 7.68 - 7.55 (m, 3H), 7.53 - 7.40 (m, 2H), 7.29 (t, J=7.5, 1H), 7.24 (s, 1H), 7.20 (t, J=7.5, 1H); Anal. calcd. for C_22_H_13_FN_4_ (%): C, 74.99; H, 3.72; N, 15.90; Found (%): C, 74.95; H, 3.75; N, 15.92.

5.3.6.7-(2-chlorophenyl)-5-(1*H*-indol-3-yl)imidazo[1,2-*a*]pyridine-8-carbonitrile (**5f**).

Yield: 69.3 %; m.p.: 296.7-300.1 ºC; MS (ESI) *m/z*: 369.3 [M+H]^+^; ^1^H NMR (400 MHz, DMSO*-d_6_*) δ (ppm) 12.22 (s, 1H), 8.29 (d, *J* = 2.7 Hz, 1H), 8.25 (s, 1H), 7.86 (s, 1H), 7.71 (d, *J* = 7.0 Hz, 2H), 7.65 - 7.53 (m, 4H), 7.28 (t, *J* = 7.5 Hz, 1H), 7.22 - 7.13 (m, 2H); Anal. calcd. for C_22_H_13_ClN_4_ (%):C, 71.64; H, 3.55; N, 15.19; Found (%): C, 71.65; H, 3.53; N, 15.14.

5.3.7.7-(3-bromophenyl)-5-(1*H*-indol-3-yl)imidazo[1,2-*a*]pyridine-8-carbonitrile (**5g**).

Yield: 69.4 %; m.p.: 321.9-324.4 ºC; MS (ESI) *m/z*: 412.9 [M+H]^+^; ^1^H NMR (400 MHz, DMSO*-d_6_*) δ (ppm) 12.14 (s, 1H), 8.28 (d, *J*=2.9, 1H), 8.14 (d, *J*=1.3, 1H), 8.04 (t, *J*=1.8, 1H), 7.87 - 7.80 (m, 2H), 7.79 - 7.73 (m, 1H), 7.59 (dt, *J*=15.7, 6.3, 3H), 7.32 - 7.25 (m, 2H), 7.20 (t, *J*=7.5, 1H); Anal. calcd. for C_22_H_13_BrN_4_ (%): C, 63.94; H, 3.17; N, 13.56; Found (%): C, 63.90; H, 3.15; N, 13.59.

5.3.8.7-(4-bromophenyl)-5-(1*H*-indol-3-yl)imidazo[1,2-*a*]pyridine-8-carbonitrile (**5h**).

Yield: 81.4 %; m.p.: 307.8-309.5 ºC; MS (ESI) *m/z*: 412.9 [M+H]^+^; ^1^H NMR (400 MHz, DMSO*-d_6_*) δ (ppm) 12.15 (1 H, s), 8.27 (1 H, d, *J* = 2.7), 8.16 (1 H, s), 7.87 - 7.73 (3 H, m), 7.61 (1 H, dd, *J* = 11.6, 8.1), 7.34 - 7.23 (2 H, m), 7.23 - 7.12 (1 H, m).; Anal. calcd. for C_22_H_13_BrN_4_ (%): C, 63.94; H, 3.17; N, 13.56; Found (%): C, 63.91; H, 3.16; N, 13.58.

5.3.9. 7-(2,4-difluorophenyl)-5-(1*H*-indol-3-yl)imidazo[1,2-*a*]pyridine-8-carbonitrile (**5i**).

Yield: 87.3 %; m.p.: 340.3-346.2 ºC; MS (ESI) *m/z*: 371.1 [M+H]^+^; ^1^H NMR (400 MHz, DMSO*-d_6_*) δ (ppm) 12.16 (s, 1H), 8.28 (d, *J*=2.8, 1H), 8.22 (d, *J*=1.2, 1H), 7.91 - 7.79 (m, 2H), 7.63 (d, *J*=8.0, 1H), 7.59 (d, *J*=8.2, 1H), 7.57 - 7.52 (m, 1H), 7.35 (td, *J*=8.4, 2.1, 1H), 7.28 (t, *J*=7.3, 1H), 7.20 (dd, *J*=16.3, 8.5, 2H); ^13^C NMR (101 MHz, DMSO*-d_6_*) δ 144.15, 138.43, 137.00, 135.53, 133.41, 128.94, 125.12, 123.23, 121.42, 119.85, 115.81, 114.33, 113.19, 112.91, 112.73, 107.53, 105.55, 105.29, 105.03, 97.09; Anal. calcd. for C_22_H_12_F_2_N_4_ (%):C, 71.35; H, 3.27; N, 15.13; Found (%): C,71.33; H, 3.24; N, 15.16.

5.3.10 .7-(3,4-difluorophenyl)-5-(1*H*-indol-3-yl)imidazo[1,2-*a*]pyridine-8-carbonitrile (**5j**).

Yield: 76.5 %; m.p.: 341.6-343.8 ºC; MS (ESI) *m/z*: 371.1 [M+H]^+^; ^1^H NMR (400 MHz, DMSO*-d_6_*) δ (ppm) 12.15 (s, 1H), 8.28 (d, *J*=2.8, 1H), 8.16 (d, *J*=1.3, 1H), 8.04 - 7.93 (m, 1H), 7.84 (d, *J*=1.2, 1H), 7.77 - 7.67 (m, 2H), 7.66 - 7.55 (m, 2H), 7.34 - 7.25 (m, 2H), 7.20 (t, *J*=7.2, 1H); Anal. calcd. for C_22_H_12_F_2_N_4_ (%): C, 71.35; H, 3.27; N, 15.13; Found (%): C,71.31; H, 3.29; N, 15.17.

5.3.11 .7-(2-chloro-4-fluorophenyl)-5-(1*H*-indol-3-yl)imidazo[1,2-*a*]pyridine-8-carbonitrile (**5k**).

Yield: 65.3%; mp: 307.9-317.3 ºC; MS (ESI) *m/z*: 387.1 [M+H]^+^; ^1^H NMR (400 MHz, DMSO*-d_6_*) δ (ppm) 12.19 (s, 1H), 8.29 (d, *J*=2.8, 1H), 8.26 (d, *J*=1.2, 1H), 7.87 (d, *J*=1.1, 1H), 7.79 (dd, *J*=8.6, 6.1, 1H), 7.74 (dd, *J*=8.9, 2.5, 1H), 7.63 (d, *J*=8.0, 1H), 7.59 (d, *J*=8.1, 1H), 7.47 (td, *J*=8.5, 2.6, 1H), 7.28 (t, *J*=7.3, 1H), 7.22 - 7.16 (m, 2H); ^13^C NMR (101 MHz, DMSO*-d_6_*) δ 164.45, 161.95, 146.16, 141.59, 139.85, 137.14, 133.64, 133.27, 131.51, 130.42, 128.70, 125.02, 123.58, 121.87, 119.83, 118.14, 117.89, 115.76, 114.09, 113.46, 106.27, 94.89; Anal.calcd. for C_22_H_12_ClFN_4_ (%): C, 68.31; H, 3.13; N, 14.48; Found (%): C, 68.36; H, 3.11; 4.93; N, 14.46.

5.3.12.7-(3-bromo-4-hydroxyphenyl)-5-(1*H*-indol-3-yl)imidazo[1,2-*a*]pyridine-8-carbonitrile (**5l**).

Yield: 69.1 %; m.p.: 309.2-312.3 ºC; MS (ESI) *m/z*: 429.0 [M+H]^+^; ^1^H NMR (400 MHz, DMSO*-d_6_*) δ (ppm) 12.12 (s, 1H), 10.85 (s, 1H), 8.25 (d, *J*=2.7, 1H), 8.08 (s, 1H), 7.98 (d, *J*=2.1, 1H), 7.79 (s, 1H), 7.68 (dd, *J*=8.4, 2.1, 1H), 7.59 (d, *J*=8.4, 2H), 7.28 (t, *J*=7.7, 1H), 7.23 (s, 1H), 7.21 - 7.12 (m, 2H); Anal. calcd. for C_22_H_13_BrN_4_O (%):C, 61.56; H, 3.05; N, 13.05;; Found (%): C, 61.51; H, 3.09; N, 13.03.

5.3.13.7-(3,5-dibromo-4-hydroxyphenyl)-5-(1*H*-indol-3-yl)imidazo[1,2-*a*]pyridine-8-carbonitrile (**5m**).

Yield: 83.8 %; m.p.: 290.5-294.2 ºC; MS (ESI) *m/z*: 506.9 [M+H]^+^; ^1^H NMR (400 MHz, DMSO*-d_6_*) δ (ppm) 12.13 (d, *J*=2.0, 1H), 10.50 (s, 1H), 8.26 (d, *J*=2.8, 1H), 8.09 (d, *J*=1.3, 1H), 8.02 (s, 2H), 7.81 (d, *J*=1.2, 1H), 7.63 - 7.54 (m, 2H), 7.31 - 7.23 (m, 2H), 7.18 (t, *J*=7.3, 1H; Anal. calcd. for C_22_H_12_Br_2_N_4_O (%):C, 52.00; H, 2.38; N, 11.03; Found (%): C, 52.05; H, 2.35; N, 11.01.

5.3.14.7-(2-bromo-4-hydroxy-5-methoxyphenyl)-5-(1H-indol-3-yl)imidazo[1,2-a]pyridine-8-carbonitrile (**5n**).

Yield: 65.2 %; m.p.: 327.7-330.0 ºC; MS (ESI) *m/z*: 459.0 [M+H]^+^; ^1^H NMR (400 MHz, DMSO*-d6*) δ (ppm) 12.12 (s, 1H), 10.04 (s, 1H), 8.25 (d, *J*=2.4, 1H), 8.06 (s, 1H), 7.80 (s, 1H), 7.58 (dd, *J*=13.0, 8.0, 3H), 7.44 (s, 1H), 7.28 (d, *J*=11.0, 2H), 7.19 (t, *J*=7.5, 1H), 3.36 (s, 3H); Anal. calcd. for C_23_H_15_BrN_4_O (%):C, 62.32; H, 3.41; N, 12.64; Found (%): C, 62.33; H, 3.40; N, 12.62.

5.3.15.7-(4-hydroxy-3,5-diisobutylphenyl)-5-(1*H*-indol-3-yl)imidazo[1,2-*a*]pyridine-8-carbonitrile (**5o**).

Yield:61.5 %; m.p.: 267.8-269.4 ºC; MS (ESI) *m/z*: 463.2 [M+H]^+^; ^1^H NMR (400 MHz, DMSO*-d_6_*) δ (ppm) 12.21 (s, 1H), 8.51 (d, *J*=2.1, 1H), 8.23 - 7.85 (m, 3H), 7.55 (s, 1H), 7.48 (t, *J*=1.1, 3H), 7.40 - 7.27 (m, 2H), 4.31 (s, 1H), 2.65 - 2.43 (m, 4H), 2.28 - 2.08 (m, 2H), 1.08 - 0.71 (m, 12H); Anal. calcd. for C_30_H_30_N_4_O (%): C, 77.89; H, 6.54; N, 12.11; Found (%): C, 78.31; H, 6.45; N, 11.82.

5.3.16. 5-(1*H*-indol-3-yl)-7-(2,3,4-trihydroxyphenyl)imidazo[1,2-a]pyridine-8-carbonitrile (**5p**).

Yield:53.5 %; m.p.: 297.8-301.4 ºC; MS (ESI) *m/z*: 383.1 [M+H]^+^; ^1^H NMR (400 MHz, DMSO*-d_6_*) δ (ppm) 12.39 (s, 1H), 8.50 (s, 1H), 8.02 (d, *J*=5.2, 2H), 7.79 (s, 1H), 7.54 (s, 1H), 7.48 (s, 1H), 7.37 - 7.23 (m, 2H), 6.92 (s, 1H), 6.40 (s, 1H), 6.34 (s, 1H), 5.57 (s, 1H), 5.05 (s, 1H); Anal. calcd. for C_22_H_14_N_4_O_3_ (%): C, 69.10; H, 3.69; N, 14.65; Found (%):C, 67.33H, 3.73; N, 15.62.

5.3.17.5-(1*H*-indol-3-yl)-7-(2,3,5-trimethoxyphenyl)imidazo[1,2-*a*]pyridine-8-carbonitrile (**5q**).

Yield: 71.3 %; m.p.: 302.7-304.8 ºC; MS (ESI) *m/z*: 423.0 [M-H]^-^; ^1^H NMR (400 MHz, DMSO*-d_6_*) δ (ppm) 12.22 (s, 1H), 8.25 (d, *J*=2.6, 1H), 8.08 (s, 1H), 7.80 (s, 1H), 7.60 (d, *J*=8.1, 1H), 7.55 (d, *J*=7.9, 1H), 7.34 (s, 1H), 7.28 (t, *J*=7.5, 1H), 7.18 (t, *J*=7.5, 1H), 7.13 (s, 2H), 3.87 (s, 6H), 3.76 (s, 3H) ;^13^C NMR (101 MHz, DMSO*-d_6_*) δ 153.56, 148.49, 142.53, 139.61, 137.16, 131.18, 130.11, 125.01, 123.27, 121.58, 120.07, 115.41, 115.34, 115.21, 113.42, 107.19, 106.66, 92.66, 60.68, 56.73.Anal.calcd. for C_25_H_20_N_4_O_3_ (%): C, 70.74; H, 4.75; N, 13.20; Found (%):C, 70.71 H, 4.76; N, 13.25.

5.3.18.5-(5-bromo-1*H*-indol-3-yl)-7-phenylimidazo[1,2-*a*]pyridine-8-carbonitrile (**5r**).

Yield: 69.7 %; m.p.: 337.9-342.4 ºC; MS (ESI) *m/z*: 410.9[M-H]^+^; ^1^H NMR (400 MHz, DMSO*-d_6_*) δ(ppm) 12.28 (s, 1H), 8.28 (d, *J*=2.8, 1H), 8.13 (d, *J*=1.3, 1H), 7.84 (d, *J*=1.2, 1H), 7.78 - 7.68 (m, 2H), 7.66 - 7.56 (m, 1H), 7.54 (d, *J*=8.7, 1H), 7.45 (dd, *J*=17.7, 9.0, 2H), 7.41 -7.29 (m, 2H), 7.21 (s, 1H); Anal. calcd. for C_22_H_13_BrN_4_ (%): C, 63.94; H, 3.17; N, 13.56; Found (%): C, 63.90; H, 3.19; N, 13.59.

5.3.19. 5-(5-bromo-1*H*-indol-3-yl)-7-(2-fluorophenyl)imidazo[1,2-*a*]pyridine-8-carbonitrile (**5s**).

Yield: 83.7 %; m.p.: 301.7-304.6 ºC; MS (ESI) *m/z*: 431.0 [M+H]^+^; ^1^H NMR (400 MHz, DMSO*-d_6_*) δ (ppm) 12.28 (s, 1H), 8.28 (d, *J*=2.7, 1H), 8.05 (s, 1H), 7.82 (d, *J*=6.1, 3H), 7.71 (s, 1H), 7.66 - 7.51 (m, 3H), 7.40 (dd, *J*=8.6, 1.6, 1H), 7.24 (s, 1H) ; Anal. calcd. for C_22_H_12_BrFN_4_ (%): C, 61.27; H, 2.80; N, 12.99; Found (%):C, 61.23; H, 2.85; N, 12.95.

5.3.20. 5-(5-bromo-1*H*-indol-3-yl)-7-(2-chlorophenyl)imidazo[1,2-*a*]pyridine-8-carbonitrile (**5t**).

Yield: 66.9 %; m.p.: 302.2-306.8 ºC; MS (ESI) *m/z*: 447.0 [M+H]^+^; ^1^H NMR (400 MHz, DMSO-*d_6_*) δ (ppm) 12.33 (s, 1H), 8.32 (d, *J*=2.7, 1H), 8.21 (s, 1H), 7.87 (s, 1H), 7.80 - 7.66 (m, 3H), 7.64 - 7.50 (m, 3H), 7.40 (dd, *J*=8.6, 1.5, 1H), 7.17 (d, *J*=15.6, 1H). Anal. calcd. for C_22_H_12_BrFN_4_ (%): C, 61.27; H, 2.80; N, 12.99; Found (%):C, 61.23; H, 2.85; N, 12.95.

5.3.21.5-(5-bromo-1*H*-indol-3-yl)-7-(4-bromophenyl)imidazo[1,2-*a*]pyridine-8-carbonitrile (**5u**).

Yield:64.9 %; m.p.: 317.2-321.8 ºC; MS (ESI) *m/z*: 491.2 [M+H]^+^; ^1^H NMR (400 MHz, DMSO) δ (ppm) 12.29 (s, 1H), 8.28 (d, *J*=2.1, 1H), 8.07 (d, *J*=1.3, 1H), 7.82 (d, *J*=8.7, 3H), 7.80 - 7.74 (m, 2H), 7.72 (d, *J*=1.7, 1H), 7.56 (d, *J*=8.7, 1H), 7.40 (dd, *J*=8.7, 1.8, 1H), 7.25 (s, 1H); Anal.calcd. for C_22_H_12_Br_2_N_4_ (%): C, 53.69; H, 2.46; N, 11.38; Found (%):C, 53.65; H, 2.49; N, 11.41.

5.3.22.5-(5-bromo-1*H*-indol-3-yl)-7-(2-chloro-4-fluorophenyl)imidazo[1,2-a]pyridine-8-carbonitrile (**5v**).

Yield: 65.8 %; m.p.: 320.8-323.2 ºC; MS (ESI) *m/z*: 465.1 [M+H]^+^; ^1^H NMR (400 MHz, DMSO-*d_6_*) δ 12.39 (s, 1H), δ = 8.59 (d, *J*=1.8, 1H), 8.04 (d, *J*=6.6, 2H), 7.88 (s, 1H), 7.78 (s, 1H), 7.66 (s, 1H), 7.53 (s, 1H), 7.48 (s, 1H), 7.38 (s, 1H), 7.22 (s, 1H). Anal. calcd. for C_22_H_11_BrClFN_4_ (%): C, 56.74; H, 2.38; N, 12.03; Found (%): C, 57.02; H, 2.46; N, 12.41.

*5.4. Preparation of 7-(2-chloro-4-fluorophenyl)-2-(chloromethyl)-5-(1H-indol-3-yl)imidazo[1,2-a] pyridine-8-carbonitrile (****6****)*

To a mixture of compound **4k** (5.0 g, 13.8 mmol) and 1,3-dichloropropan-2-one (18.2 g, 144.1 mmol) in ethanol (50 mL) were added and refluxed for 10 h. After the completion of the reaction indicated by TLC, the solvent was evaporated and then the residue was washed with ethyl ether (50mL) for 2h to give the corresponding crude product in 64.7% yield. MS (ESI) *m/z*: 435.1 [M+H]^+^.

*5.5. General procedure for the preparation of compounds (****7a - 7h****).*

A stirring mixture of **6** (0.5 g, 1.2 mmol) and an appropriate *N*-aliphatic amines (1.8 mmol) in ethanol (15 mL) was stirred at reflux for 5 h. After cooling to room temperature, the solvent was evaporated in *vacuum* to afford yellow residue. The formed precipitate was dissolved in DMF and purified on a silica gel column (petroleum ether/ ethyl acetate, 3:1) to give pure target product **7a** - **7h**.

5.5.1.7-(2-chloro-4-fluorophenyl)-5-(1*H*-indol-3-yl)-2-((methylamino)methyl)imidazo[1,2-a]pyridine-8-carbonitrile (**7a**).

Yield: 83.3 %; m.p.: 219.5-223.5 ºC; MS (ESI) *m/z*: 430.1 [M+H]^+^; ^1^H NMR (400 MHz, DMSO*-d_6_*) δ (ppm) 12.21 (s, 1H), 8.24 (s, 1H), 8.18 (s, 1H), 7.78 - 7.70 (m, 2H), 7.63 - 7.56 (m, 2H), 7.45 (td, *J* = 8.5, 2.6 Hz, 1H), 7.27 (t, *J* = 7.5 Hz, 1H), 7.20 - 7.13 (m, 2H), 3.99 (s, 2H), 2.40 (d, *J* = 10.1 Hz, 3H); Anal. calcd. forC_27_H_23_ClFN_5_ (%): C, 68.71; H, 4.91; N, 14.84; Found (%): C, 68.70; H, 4.93; N, 14.82.

5.5.2.7-(2-chloro-4-fluorophenyl)-2-((ethylamino)methyl)-5-(1*H*-indol-3-yl)imidazo[1,2-a]pyridine-8-carbonitrile (**7b**).

Yield: 87.4 %; m.p.: 170.7-172.6 ºC ; MS (ESI) *m/z*: 444.1 [M+H]^+^; ^1^H NMR (400 MHz, DMSO-*d*_6_) δ (ppm) 12.21 (s, 1H), 8.26 (s, 1H), 8.14 (s, 1H), 7.75 (ddd, *J*=11.4, 8.8, 4.4, 3H), 7.61 (t, *J*=7.4, 2H), 7.46 (td, *J* =8.5, 2.6, 1H), 7.28 (t, *J* =7.9, 1H), 7.19 (dd, *J* =12.6, 4.8, 2H), 7.14 (s, 1H), 3.96 (s, 2H), 2.68 (q, *J* =7.1, 1H), 1.06 (t, *J* =7.1, 3H) ; Anal. calcd. for C_25_H_19_ClFN_5_ (%): C, 67.64; H, 4.31; N, 15.78; Found (%):C, 67.62; H, 4.34; N, 15.83.

5.5.3.7-(2-chloro-4-fluorophenyl)-5-(1*H*-indol-3-yl)-2-((isopropylamino)methyl)imidazo[1,2-a]pyridine-8-carbonitrile (**7c**).

Yield:83.9%; m.p.: 222.2-225.3 ºC; MS (ESI) *m/z*: 458.2 [M+H]^+^; ^1^H NMR (400 MHz, DMSO*-d_6_*) δ (ppm) 12.25 (s, 1H), 8.24 (s, 1H), 8.12 (s, 1H), 7.73 (ddd, *J* = 11.4, 8.8, 4.4 Hz, 2H), 7.59 (dd, *J* = 8.0, 5.4 Hz, 2H), 7.46 - 7.41 (m, 1H), 7.26 (t, *J* = 7.3 Hz, 1H), 7.16 (dd, *J* = 11.4, 4.4 Hz, 1H), 7.12 (d, *J* = 5.1 Hz, 1H), 3.91 (s, 2H), 2.83 (dt, *J* = 12.4, 6.3 Hz, 1H), 1.02 (d, *J* = 6.2 Hz, 6H); Anal. calcd. for C_26_H_21_ClFN_5_ (%): C, 68.19; H, 4.62; N, 15.29; Found (%):C, 68.15; H, 4.64; N, 15.31.

5.5.4.7-(2-chloro-4-fluorophenyl)-2-((dimethylamino)methyl)-5-(1*H*-indol-3-yl)imidazo[1,2-a]pyridine-8-carbonitrile (**7d**).

Yield: 78.3%; m.p.: 175.4-178.4 ºC; MS (ESI) *m/z*: 444.1 [M+H]^+^; ^1^H NMR (400 MHz, DMSO*-d_6_*) δ 12.28 (s, 1H), 8.25 (s, 1H), 8.07 (s, 1H), 7.78 - 7.68 (m, 2H), 7.59 (d, *J* = 7.9 Hz, 2H), 7.47 - 7.40 (m, 1H), 7.28 - 7.22 (m, 1H), 7.15 (dd, *J* = 14.6, 7.2 Hz, 2H), 3.68 (s, 2H), 2.26 (s, 6H); Anal. calcd. for C_25_H_19_ClFN_5_ (%): C, 67.64; H, 4.31; N, 15.78; Found (%):C, 67.61; H, 4.33; N, 15.80.

5.5.5.7-(2-chloro-4-fluorophenyl)-2-((diethylamino)methyl)-5-(1*H*-indol-3-yl)imidazo[1,2-a]pyridine-8-carbonitrile (**7e**).

Yield: 69.5%; m.p.: 181.2-184.5 ºC; MS (ESI) *m/z*: 472.2 [M+H]^+^; ^1^H NMR (400 MHz, DMSO*-d_6_*) δ (ppm) 12.27 (s, 1H), 8.23 (s, 1H), 7.99 (s, 1H), 7.79 - 7.72 (m, 2H), 7.59 (dd, *J* = 17.6, 8.1 Hz, 2H), 7.46 (td, *J* = 8.5, 2.6 Hz, 1H), 7.31 - 7.27 (m, 1H), 7.17 (t, *J* = 7.4 Hz, 1H), 7.13 (s, 1H), 3.78 (s, 2H), 2.57 - 2.51 (m, 4H), 1.00 (t, *J* = 7.1 Hz, 6H); Anal. calcd. for C_27_H_23_ClFN_5_ (%): C, 68.71; H, 4.91; N, 14.84; Found (%): C, 68.70; H, 4.93; N, 14.82.

5.5.6.7-(2-chloro-4-fluorophenyl)-5-(1*H*-indol-3-yl)-2-((4-methylpiperidin-1-yl)methyl)imidazo[1,2-a]pyridine-8-carbonitrile (**7f)**.

Yield: 63.5%; m.p.: 185.7-187.9 ºC; MS (ESI) *m/z*: 498.2 [M+H]^+^; ^1^H NMR (400 MHz, DMSO*-d_6_*) δ (ppm) 12.18 (s, 1H), 8.24 (s, 1H), 7.99 (s, 1H), 7.74 (ddd, J = 11.5, 8.8, 4.3 Hz, 2H), 7.57 (dd, *J* = 8.0, 4.6 Hz, 2H), 7.44 (td, *J* = 8.5, 2.6 Hz, 1H), 7.26 (t, *J* = 7.8 Hz, 1H), 7.15 (t, *J* = 7.5 Hz, 1H), 7.11 (s, 1H), 3.63 (s, 2H), 2.87 (d, *J* = 11.3 Hz, 2H), 1.98 (t, *J* = 10.7 Hz, 2H), 1.53 (d, *J* = 11.2 Hz, 2H), 1.27 (d, *J* = 4.2 Hz, 1H), 1.09 (dt, *J* = 20.7, 7.4 Hz, 2H), 0.84 (d, *J* = 6.5 Hz, 3H); Anal. calcd. for C_29_H_25_ClFN_5_ (%):C, 69.94; H, 5.06; N, 14.06; Found (%): C, 69.91; H, 5.03; N, 14.05.

5.5.7.7-(2-chloro-4-fluorophenyl)-5-(1*H*-indol-3-yl)-2-(pyrrolidin-1-ylmethyl)imidazo[1,2-a]pyridine-8-carbonitrile (**7g**).

Yield: 86.1 %; m.p.: 195.0-199.7 ºC; MS (ESI) *m/z*: 470.2 [M+H]^+^; ^1^H NMR (400 MHz, DMSO*-d_6_*) δ (ppm)12.17 (s, 1H), 8.25 (s, 1H), 8.01 (s, 1H), 7.74 (ddd, *J* = 11.4, 8.8, 4.3 Hz, 2H), 7.58 (d, *J* = 8.6 Hz, 2H), 7.46 - 7.41 (m, 1H), 7.28 - 7.24 (m, 1H), 7.17 (d, *J* = 8.0 Hz, 1H), 7.11 (s, 1H), 3.77 (d, *J* = 14.2 Hz, 2H), 2.50 - 2.46 (m, 4H), 1.67 (s, 4H); Anal. calcd. for C_27_H_21_ClFN_5_ (%):C, 69.01; H, 4.50; N, 14.90; Found (%): C, 69.04; H, 4.51; N, 14.92.

5.5.8.7-(2-chloro-4-fluorophenyl)-5-(1*H*-indol-3-yl)-2-(morpholinomethyl)imidazo[1,2-a]pyridine-8-carbonitrile (**7h**).

Yield: 58.7 %; m.p.: 201.3-203.5 ºC; MS (ESI) *m/z*: 486.1 [M+H]^+^; ^1^H NMR (400 MHz, DMSO*-d_6_*) δ (ppm) 12.17 (s, 1H), 8.24 (d, *J* = 2.0 Hz, 1H), 8.02 (s, 1H), 7.82 (dd, *J* = 15.2, 8.6 Hz, 1H), 7.55 (dd, *J* = 21.4, 8.8 Hz, 2H), 7.33 (t, *J* = 7.4 Hz, 1H), 7.26 (d, *J* = 7.5 Hz, 1H), 7.21 - 7.13 (m, 1H), 3.66 (s, 1H), 3.54 (s, 2H). Anal. calcd. for C_27_H_21_ClFN_5_O (%): C, 66.74; H, 4.36; N, 14.41; Found (%): C, 66.71; H, 4.37; N, 14.43;

Mass spectra (MS), ^1^H NMR (400 MHz, DMSO*-d_6_*) and ^13^C NMR (101 MHz, DMSO-d6)

**1. 5a**


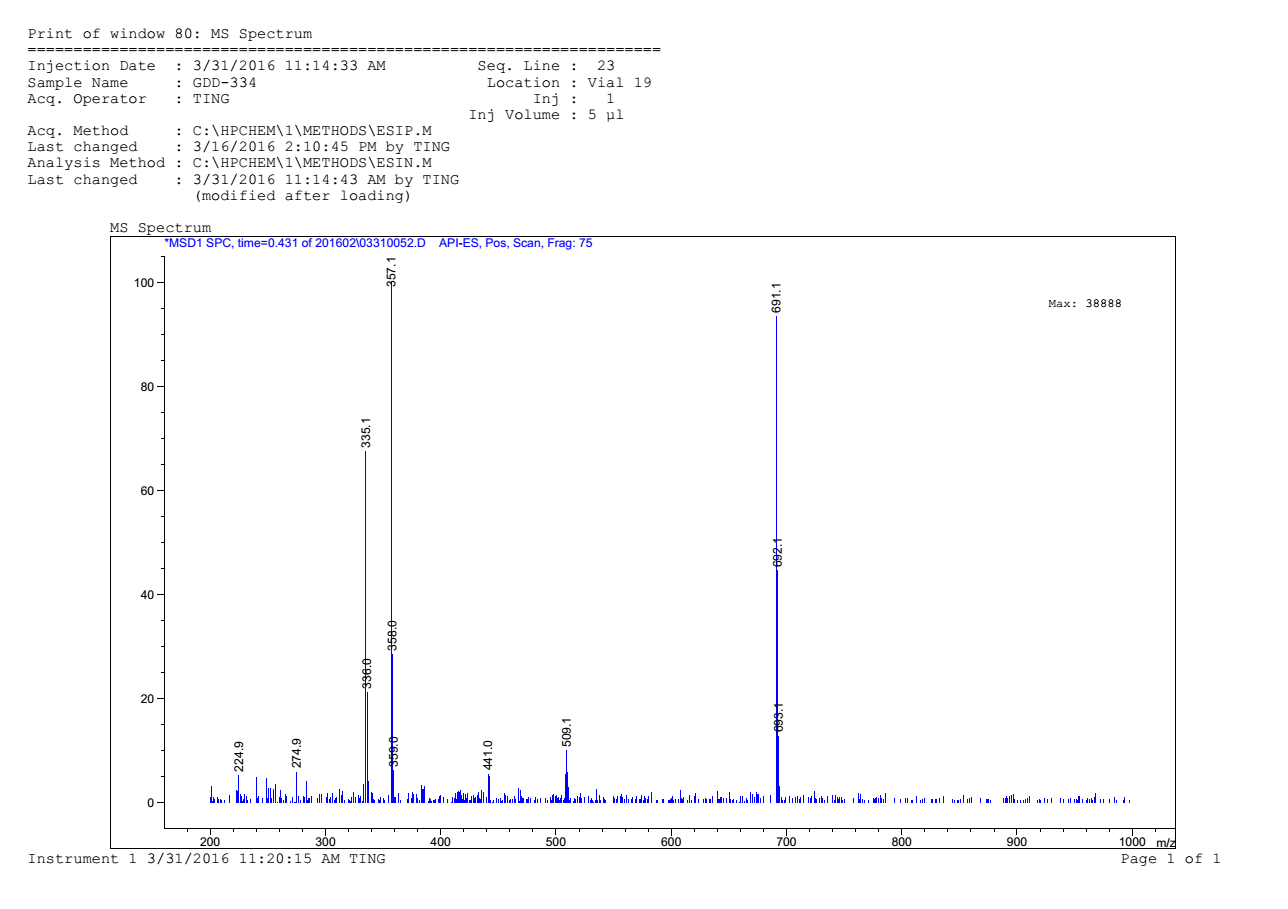


**2. 5c**

**
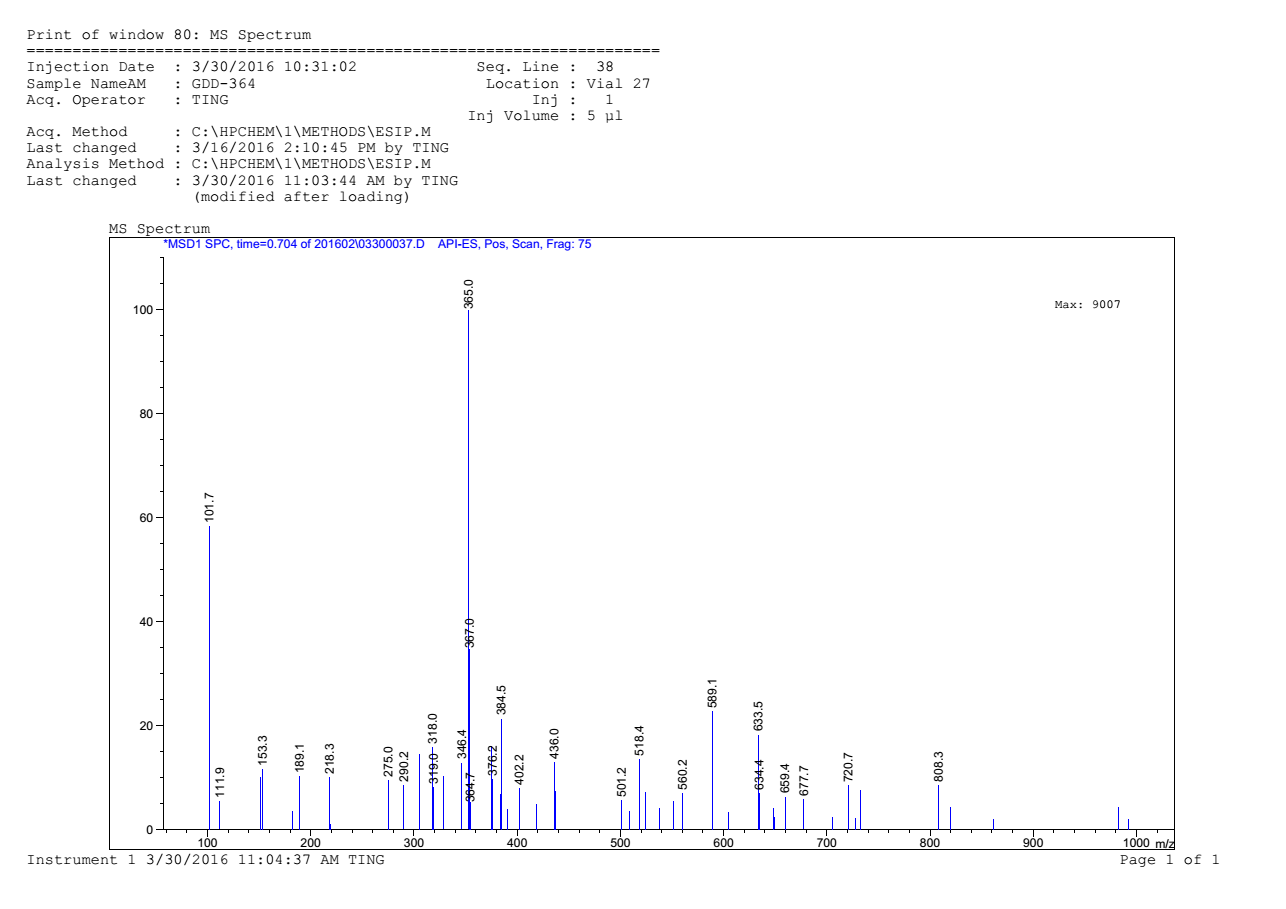
**

**3. 5d**

**
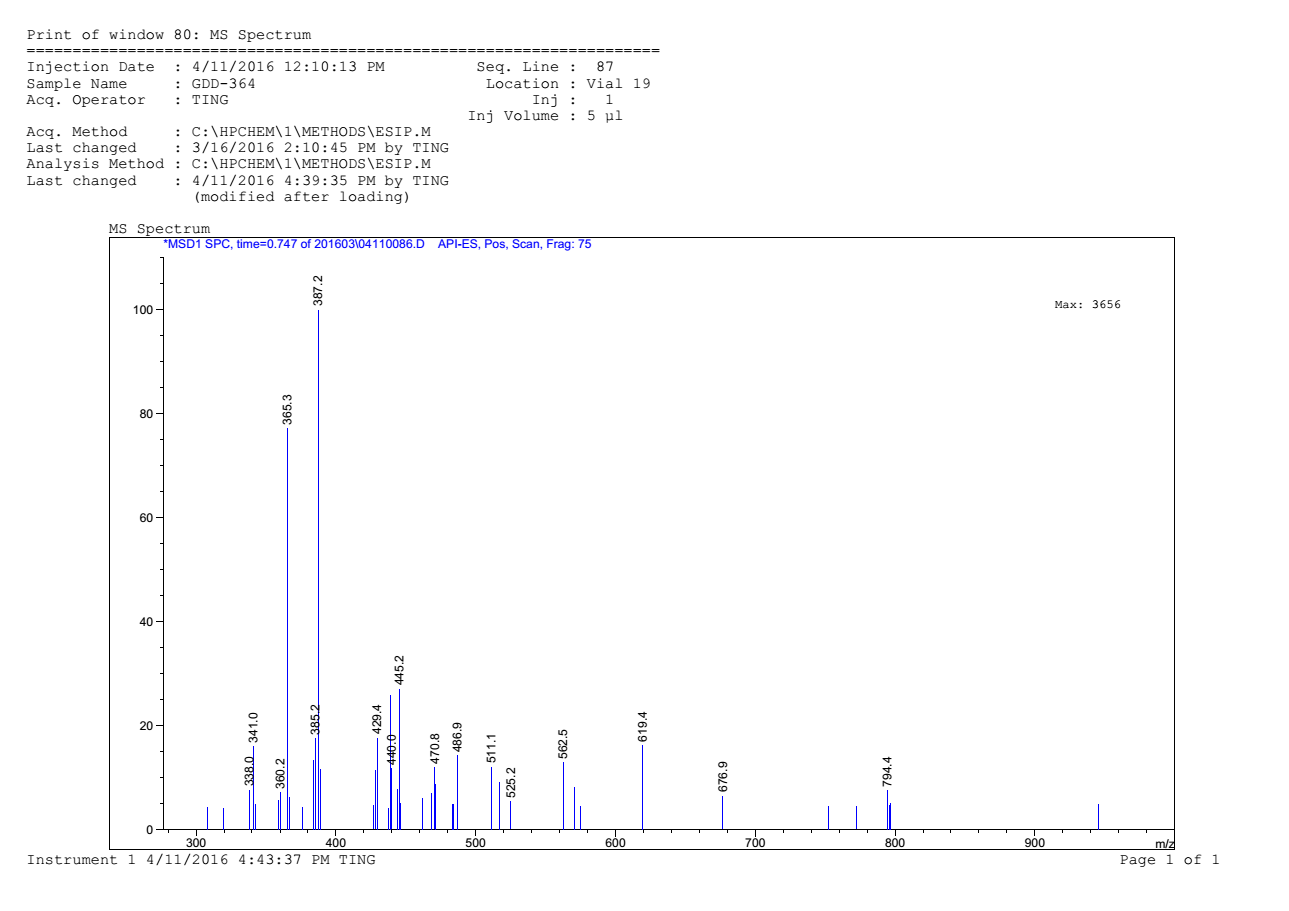
**

**4. 5e**


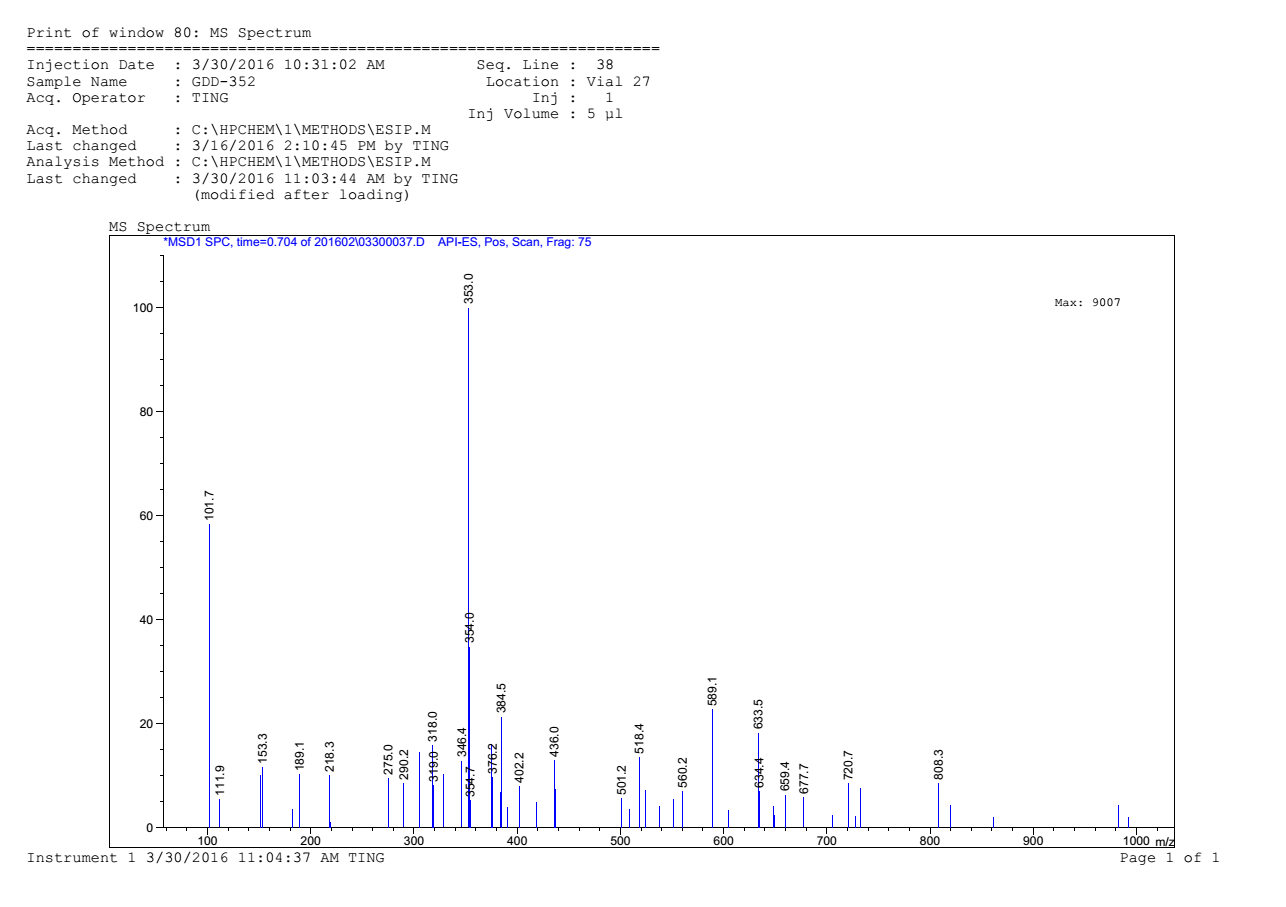


**5. 5f**

**
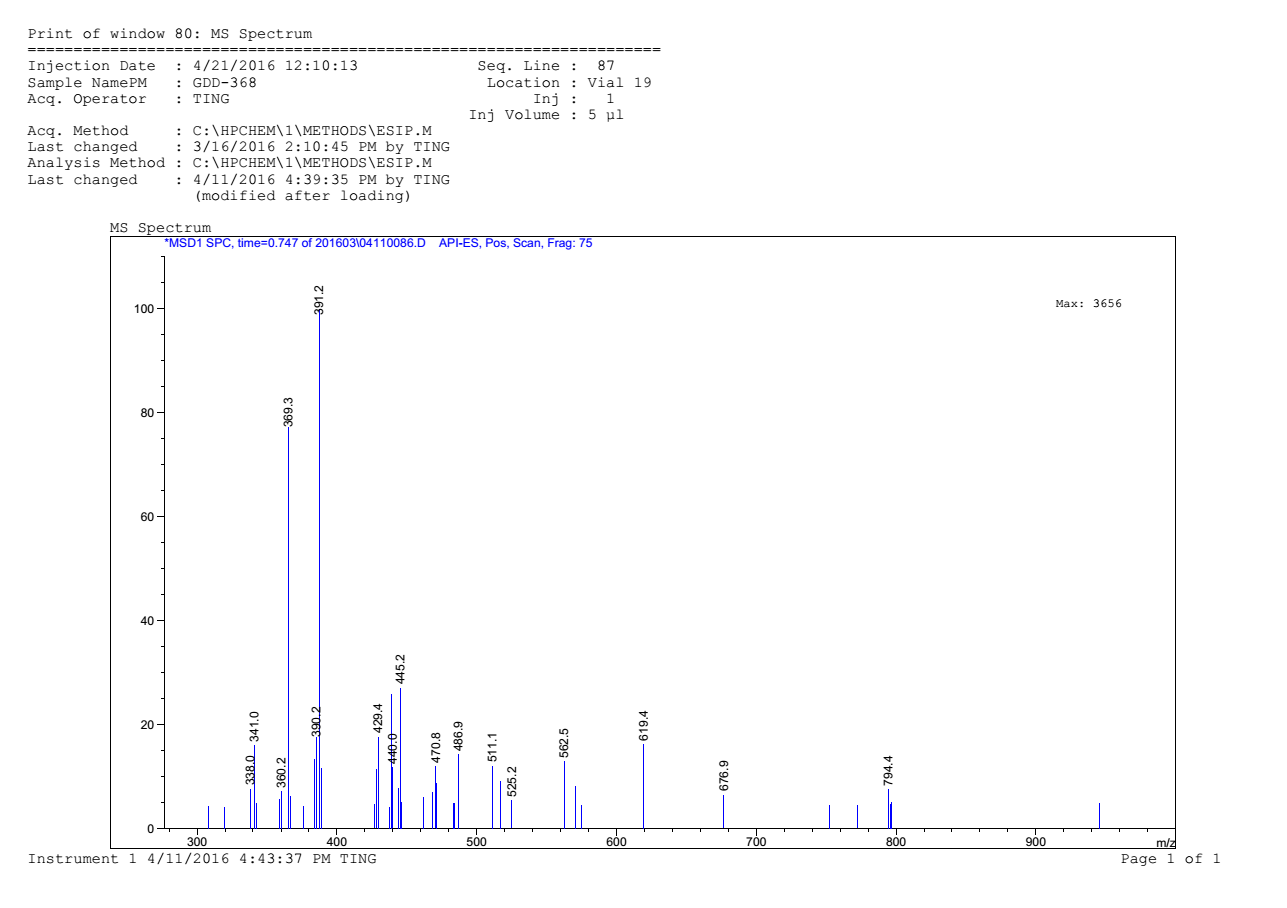
**

**6. 5g**

**
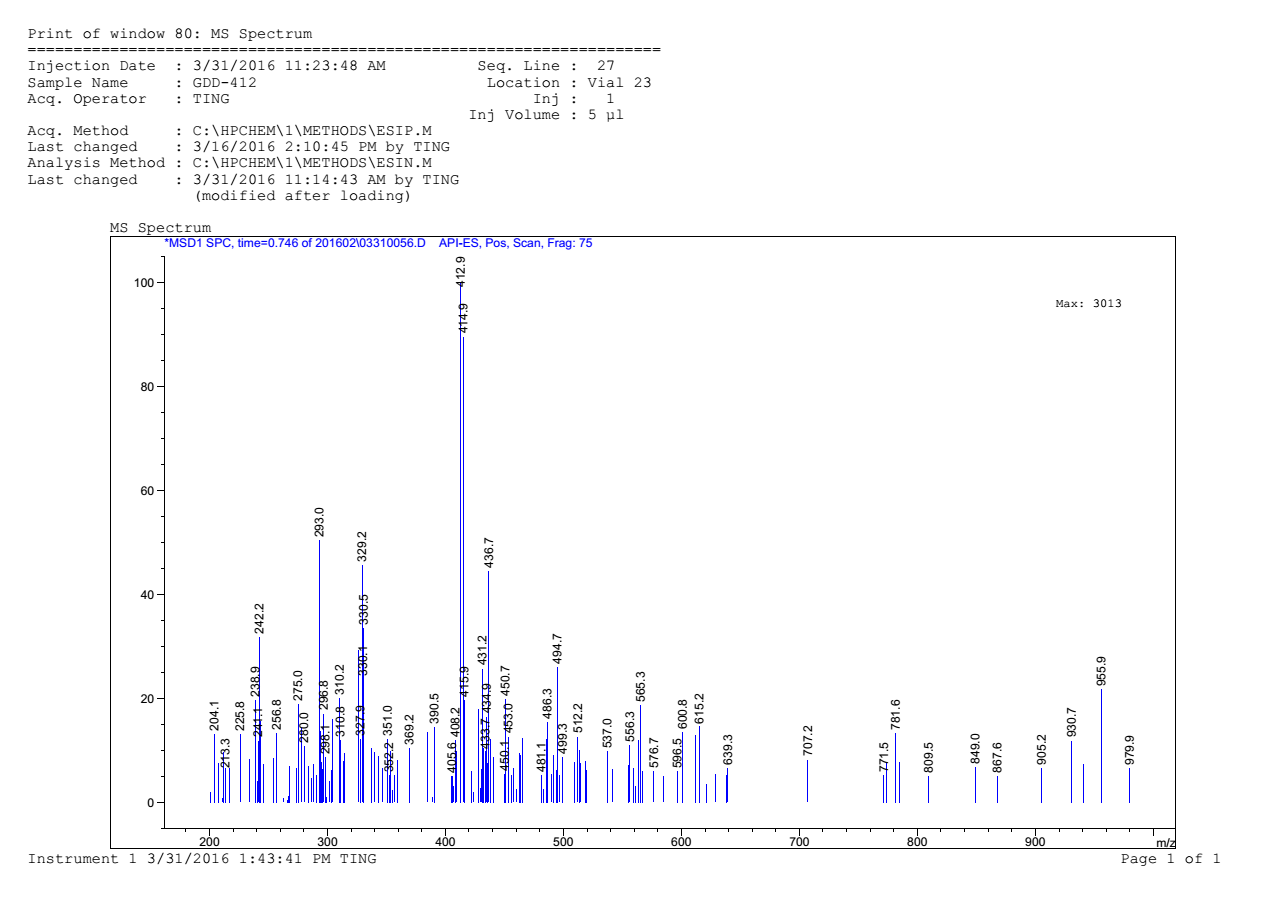
**

**7. 5h**

**
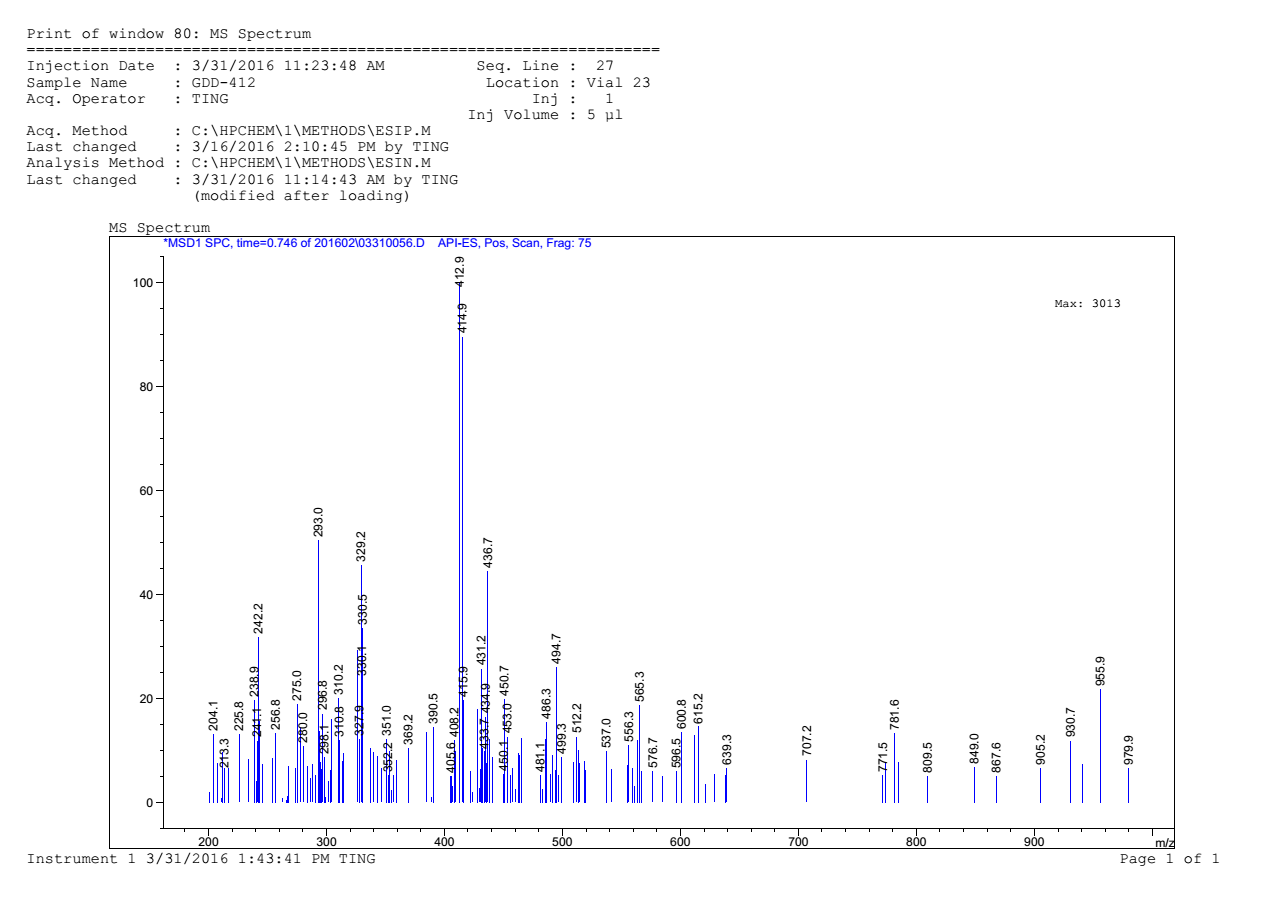
**

**8. 5i**

**
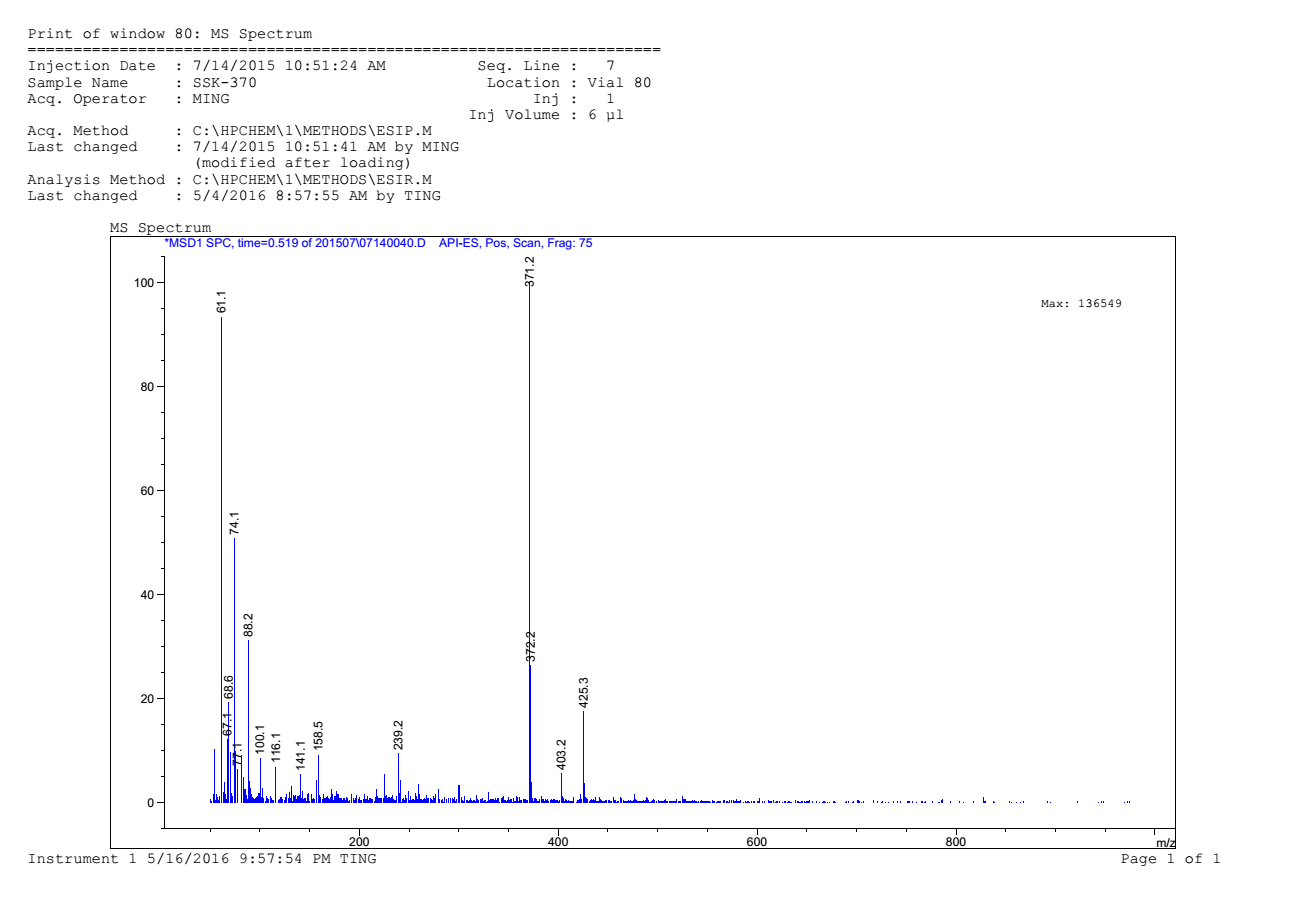
**

**9. 5j**

**10. 5k**

**
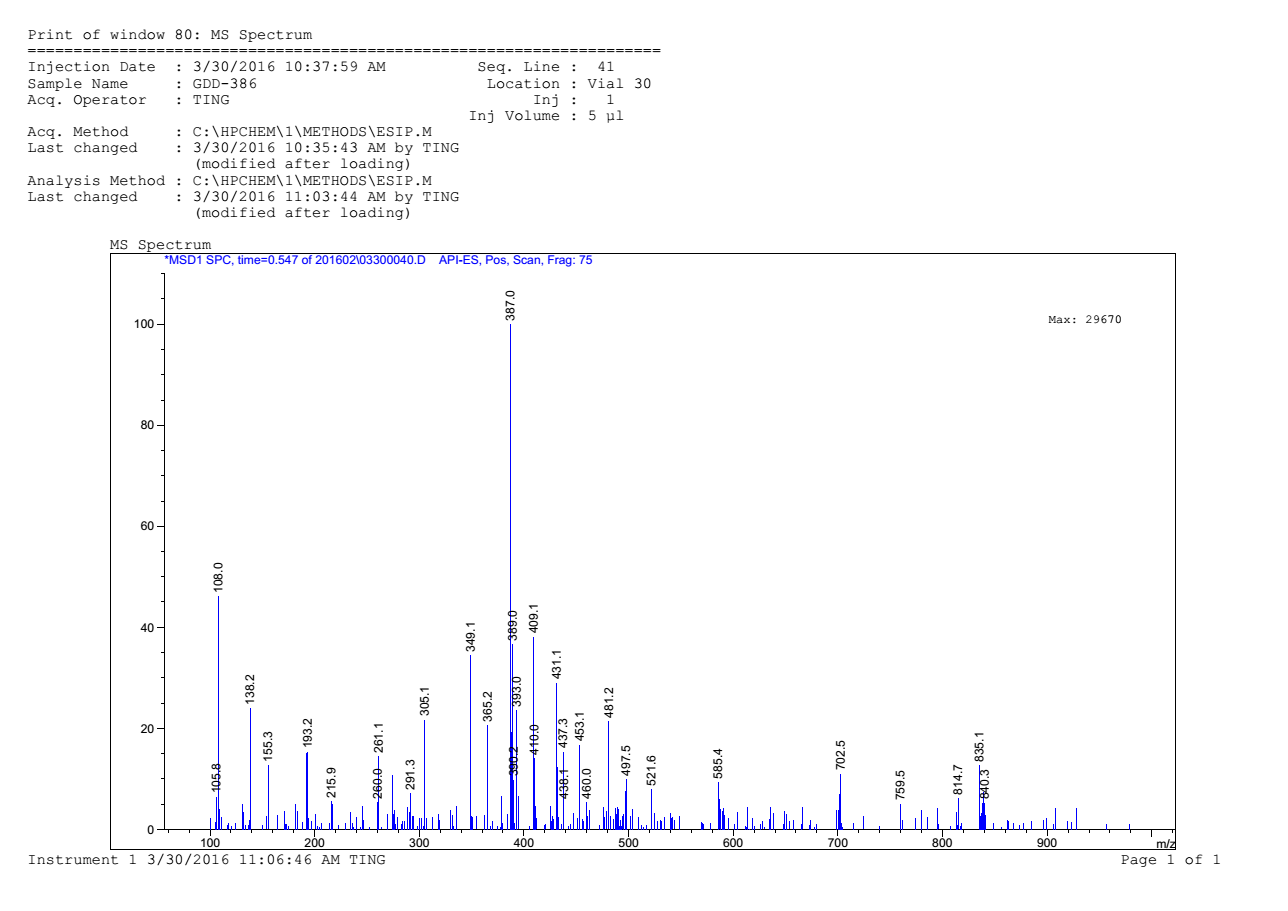
**

**11. 5l**

**
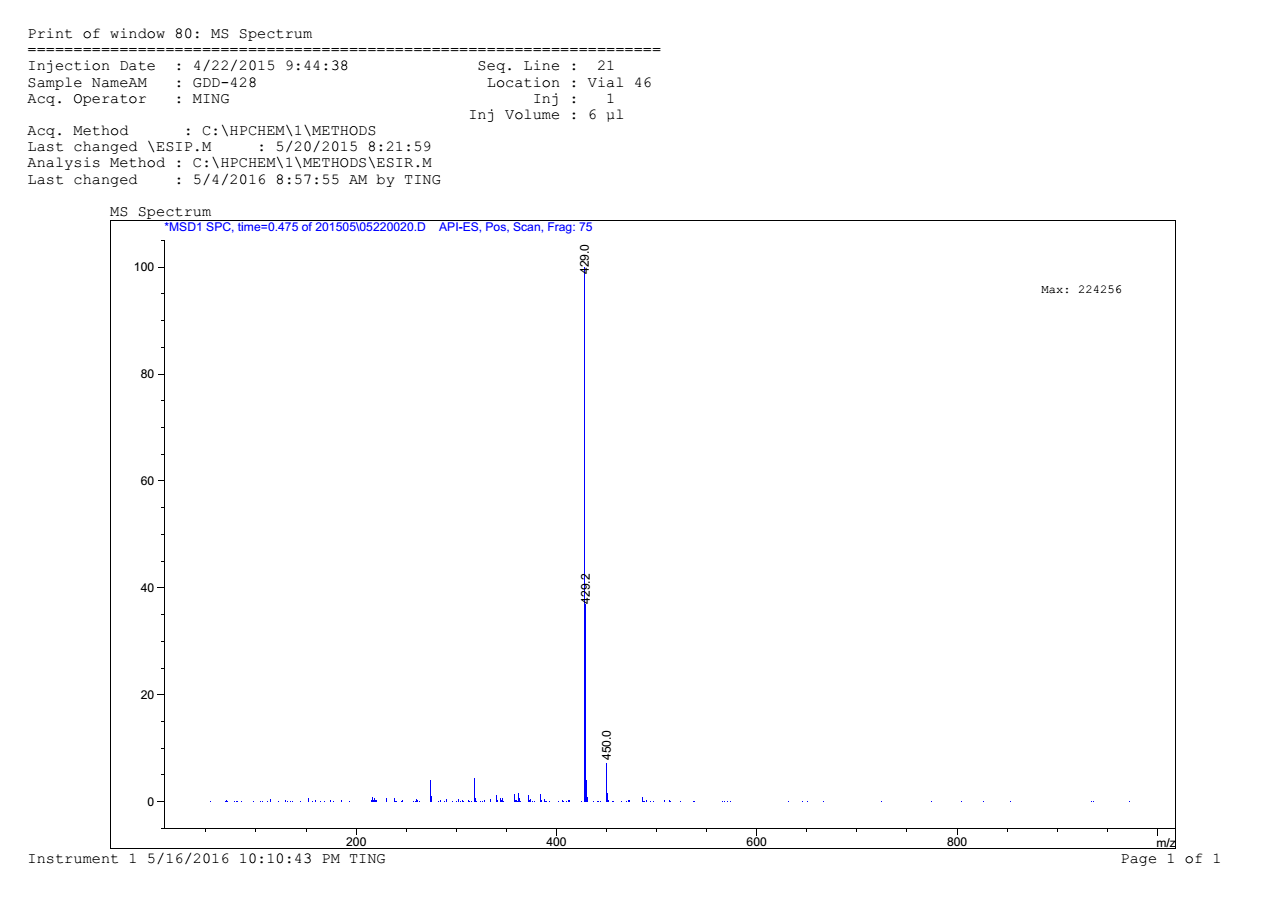
**

**12. 5m**

**
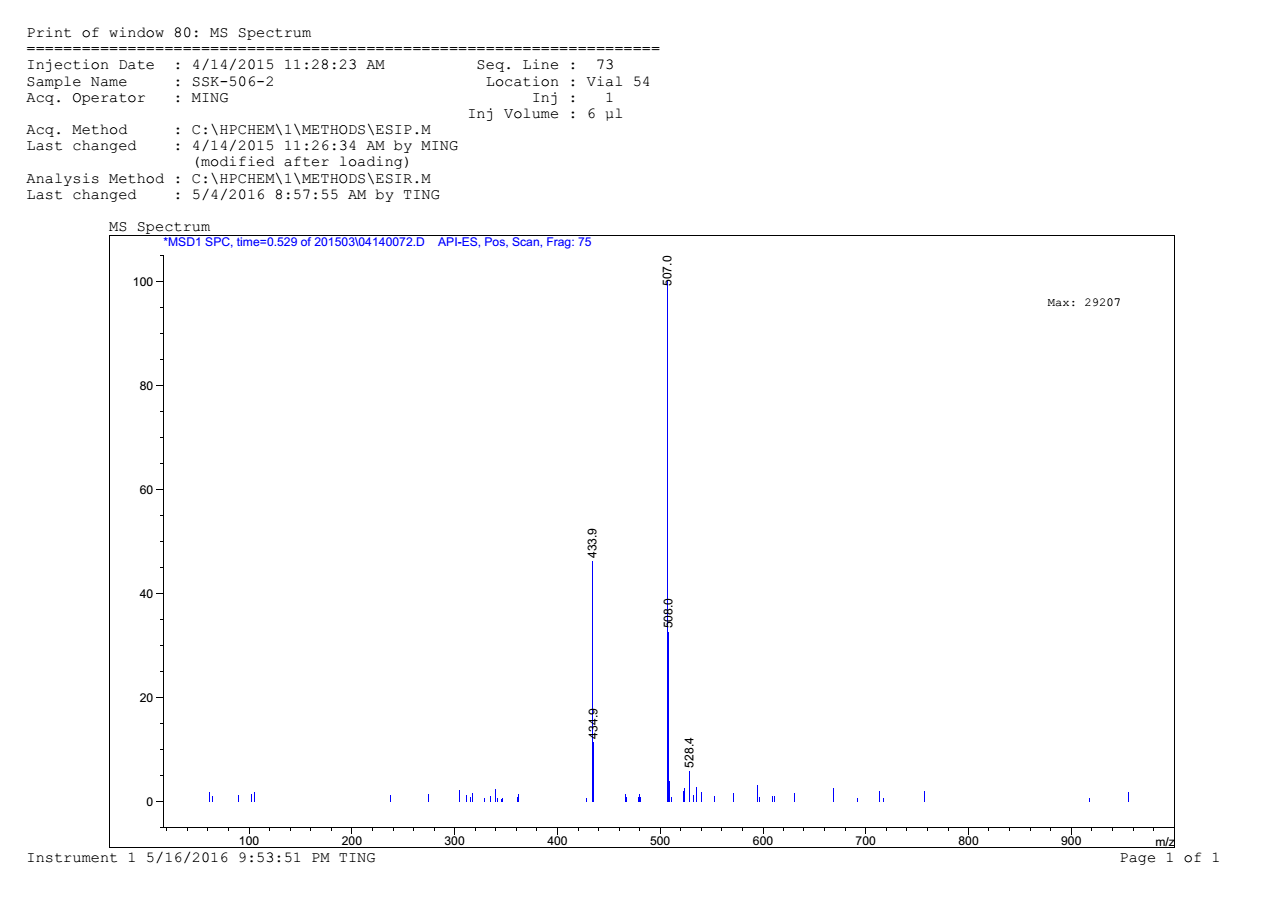
**

**13. 5n**

**
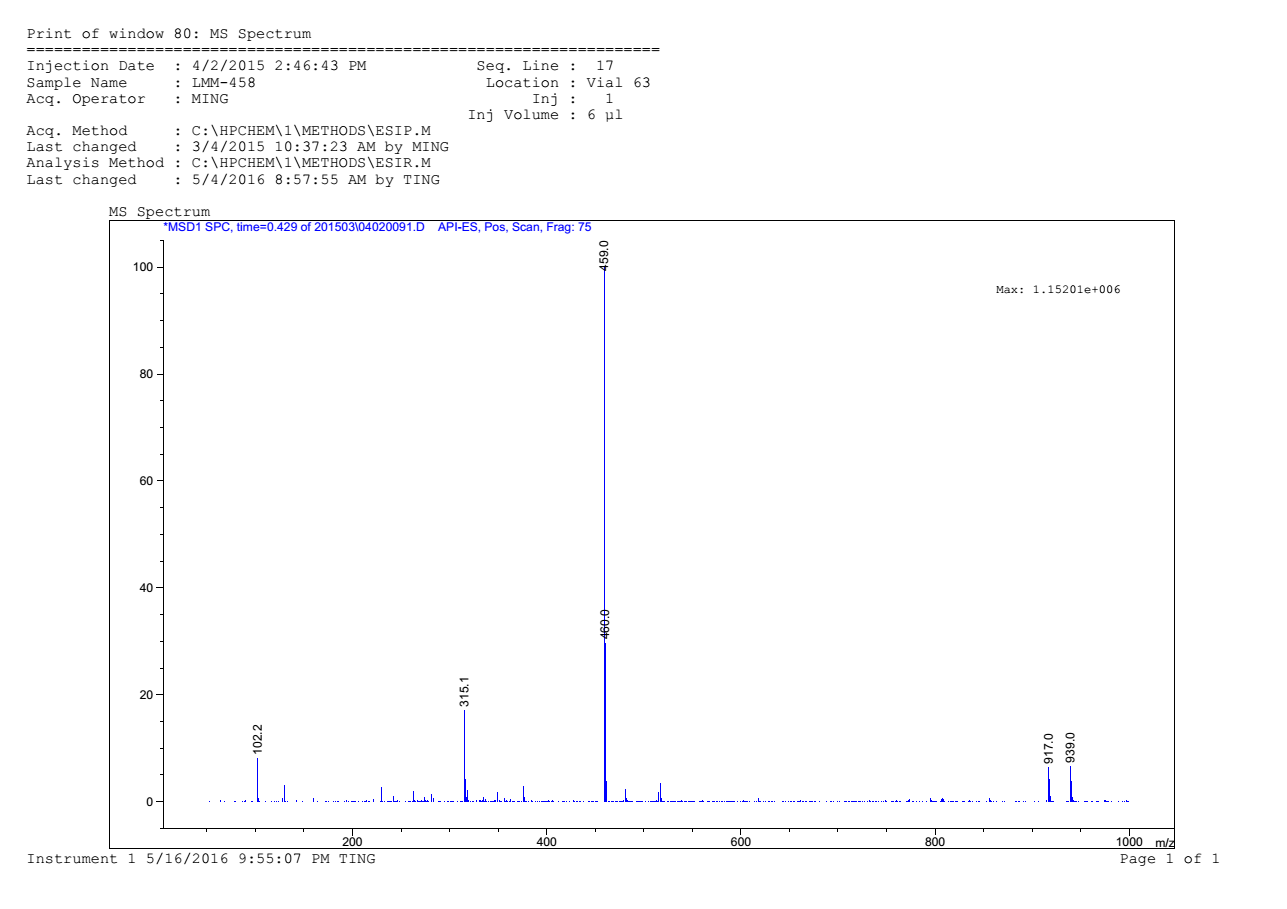
**

**14. 5q**

**
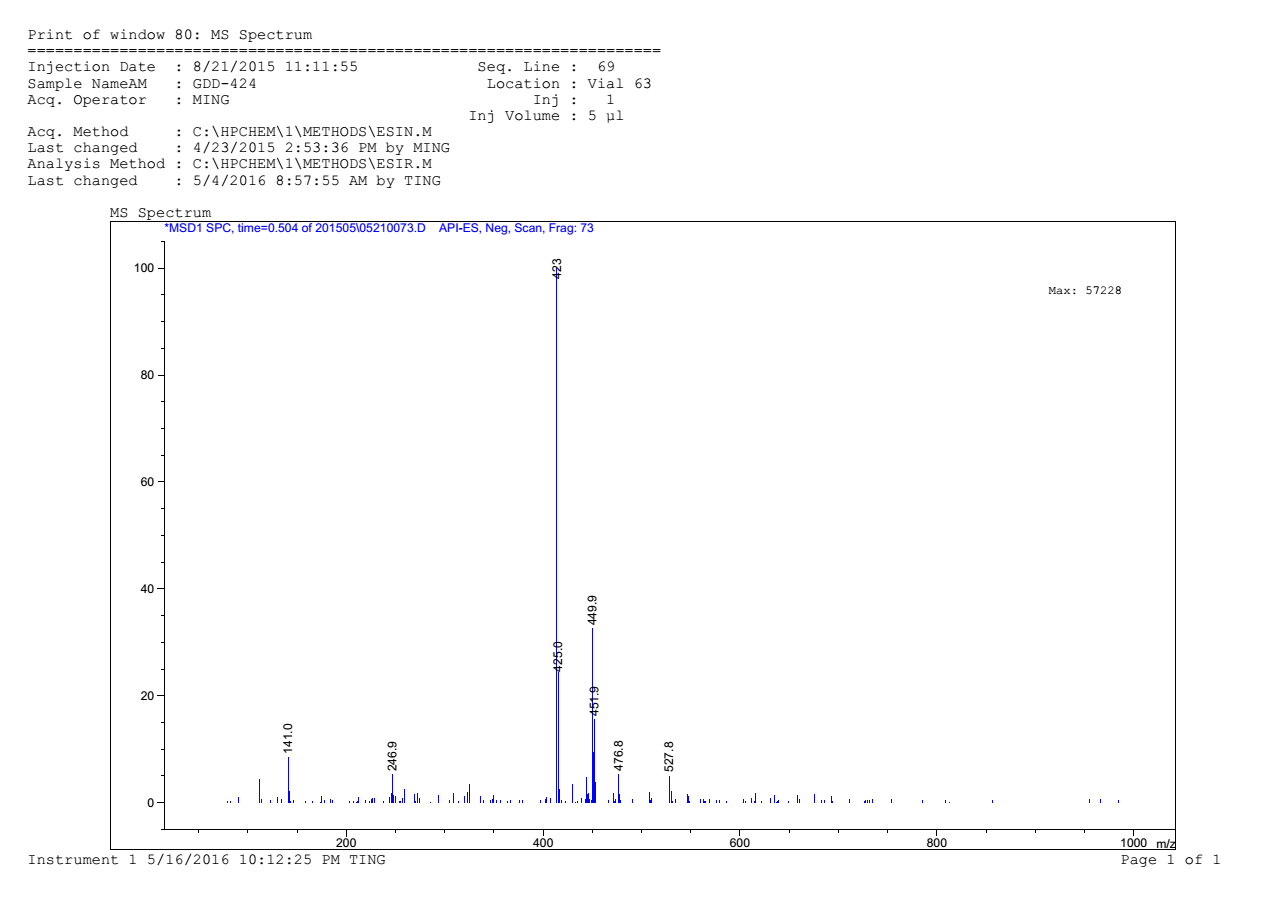
**

**15. 5r**

**
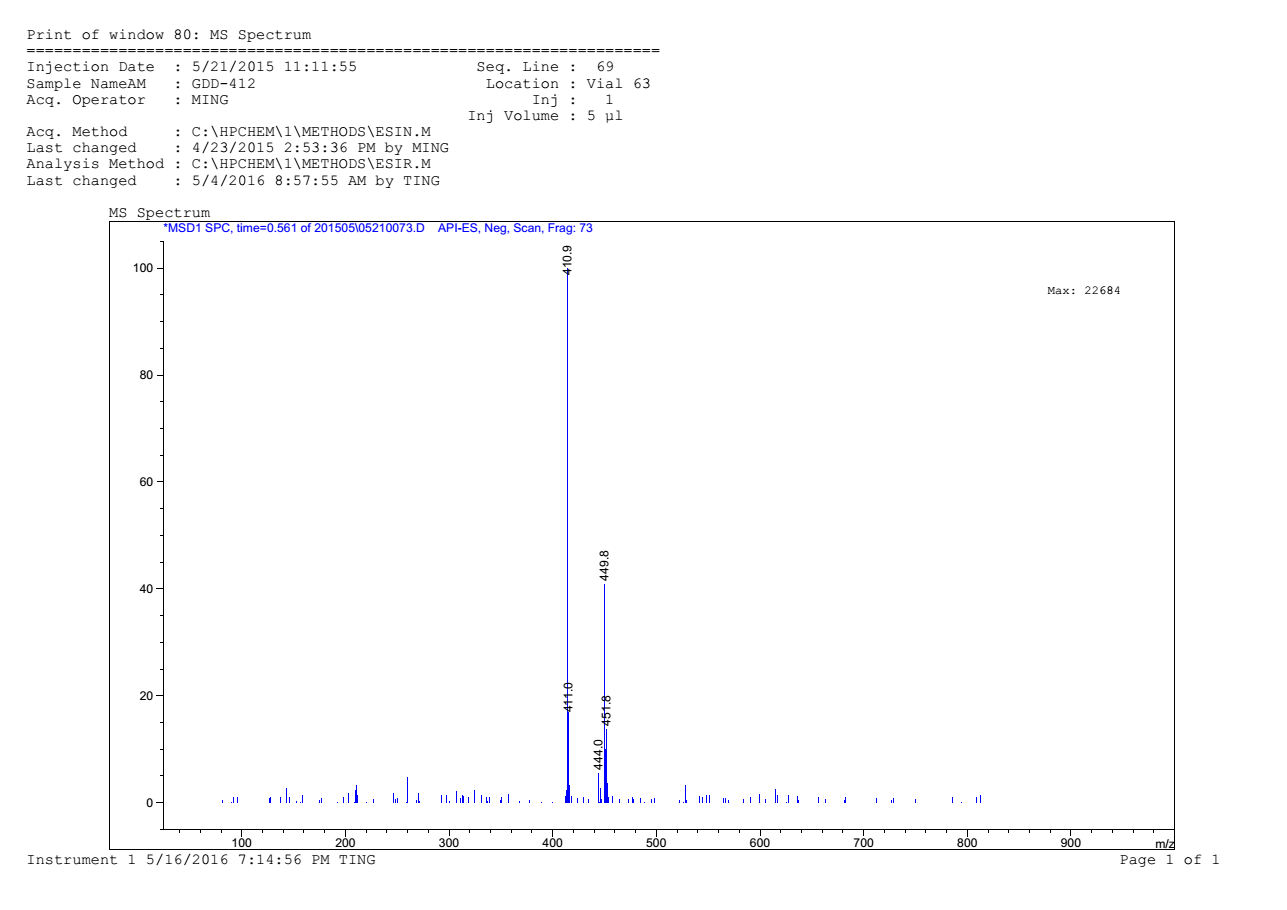
**

**16. 5s**

**
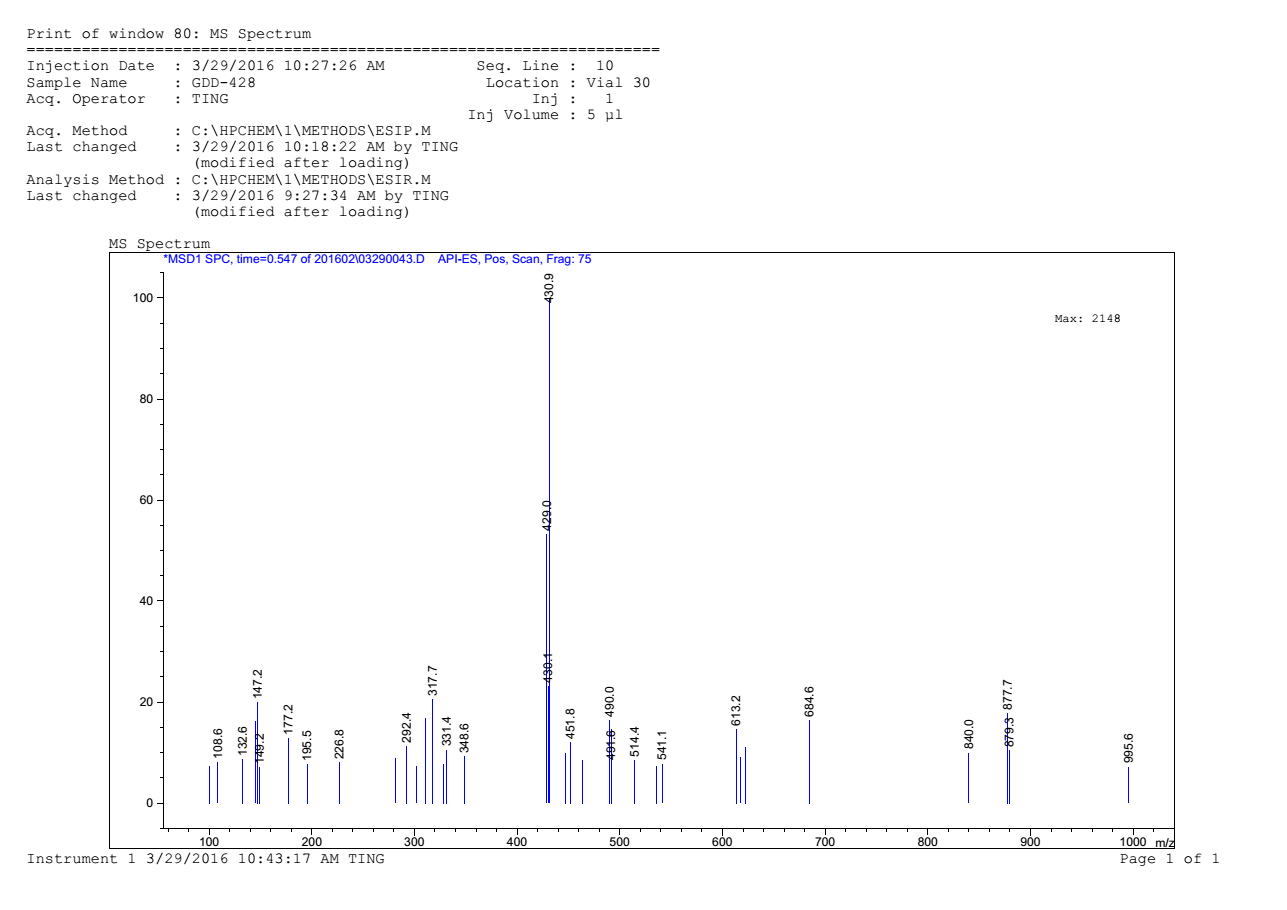
**

**17. 5t**

**
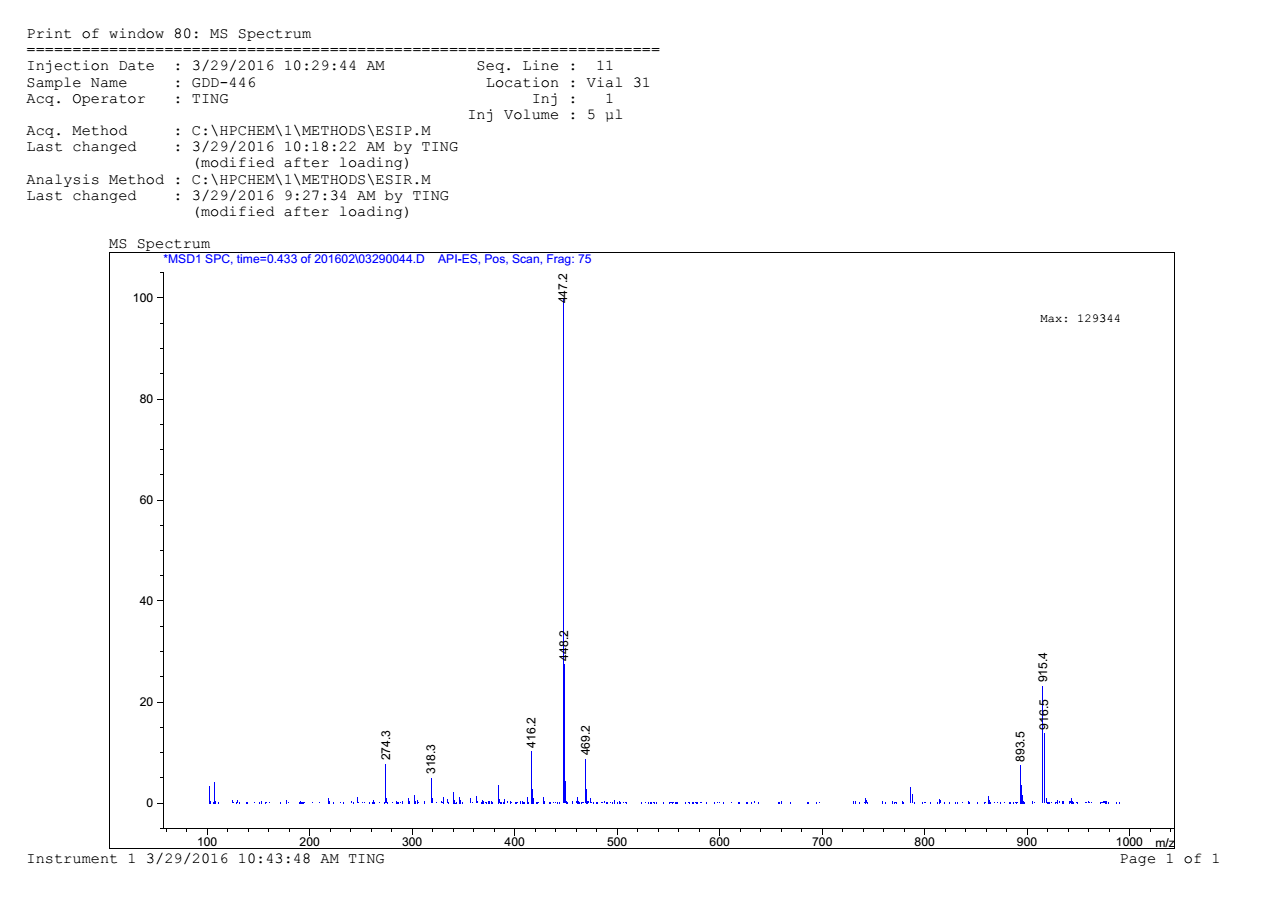
**

**18. 5u**

**
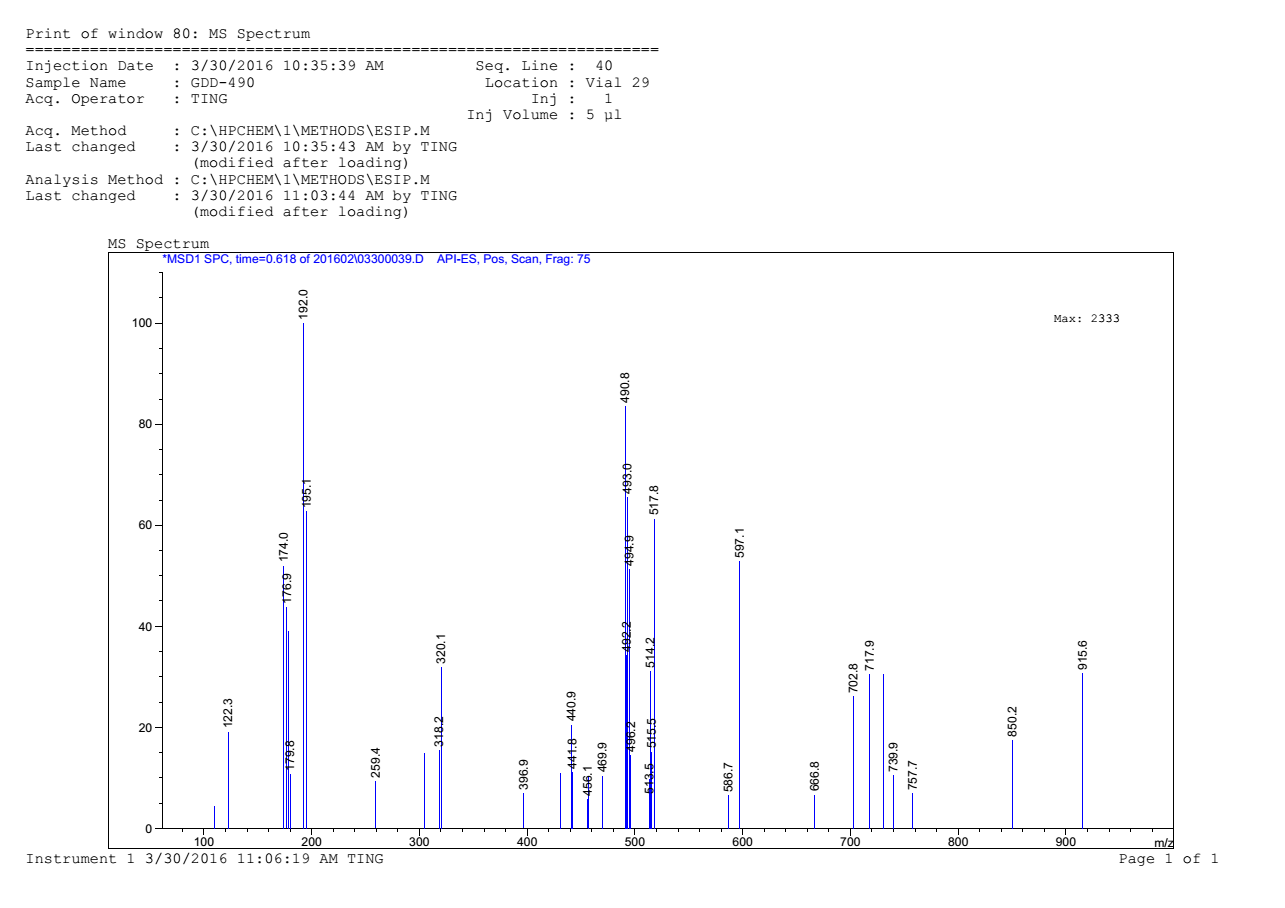
**

**19. 7a**

**
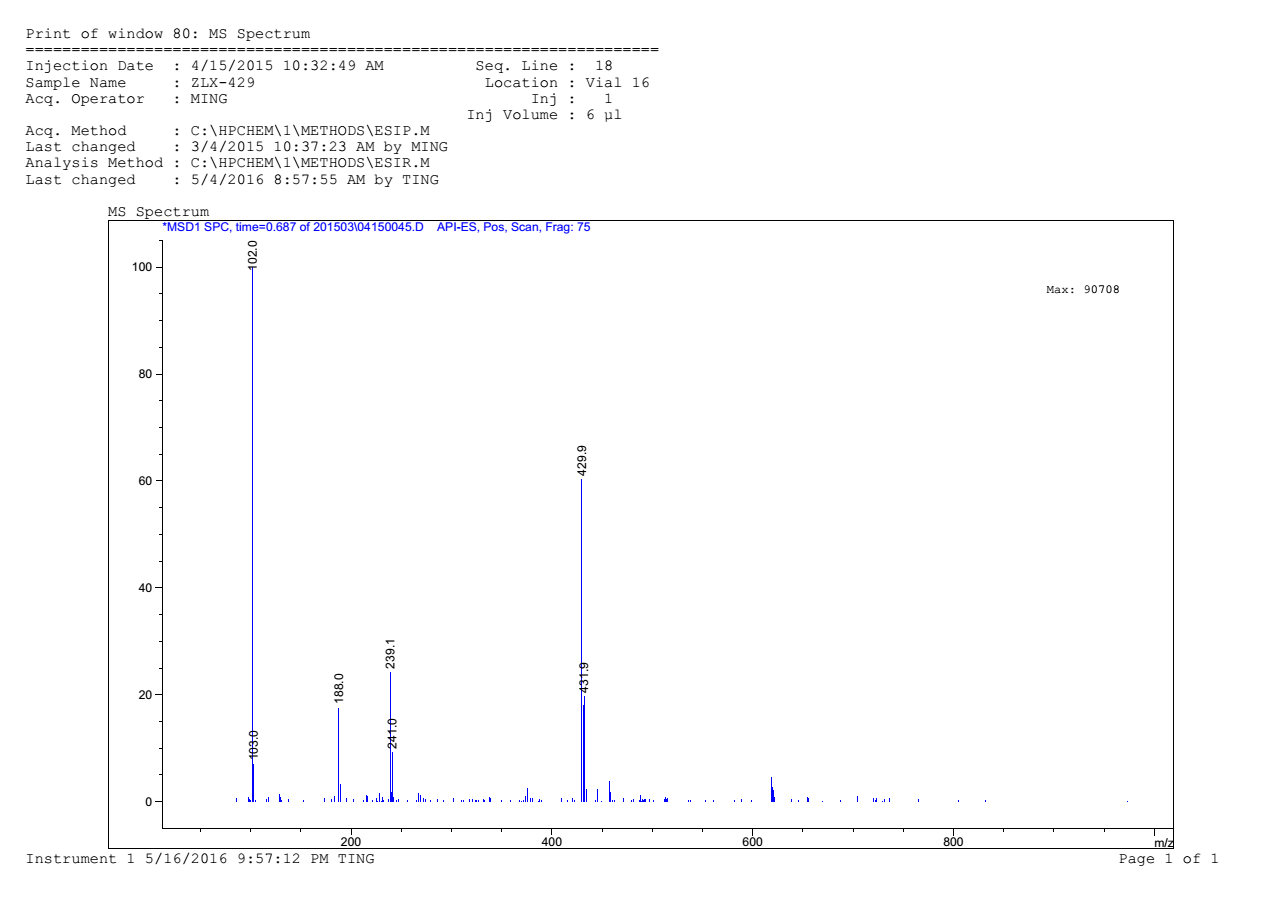
**

**20. 7b**

**
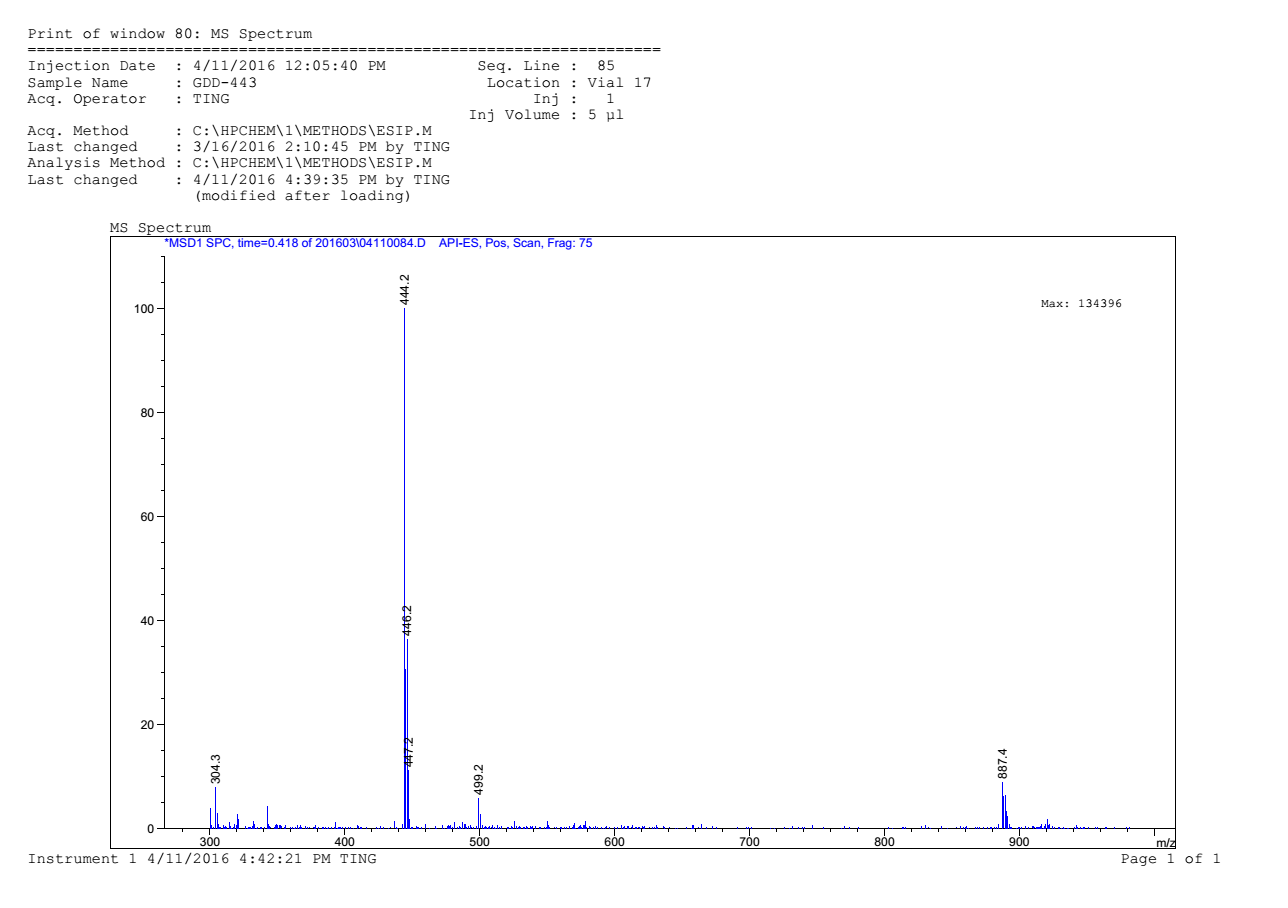
**

**21. 7c**

**
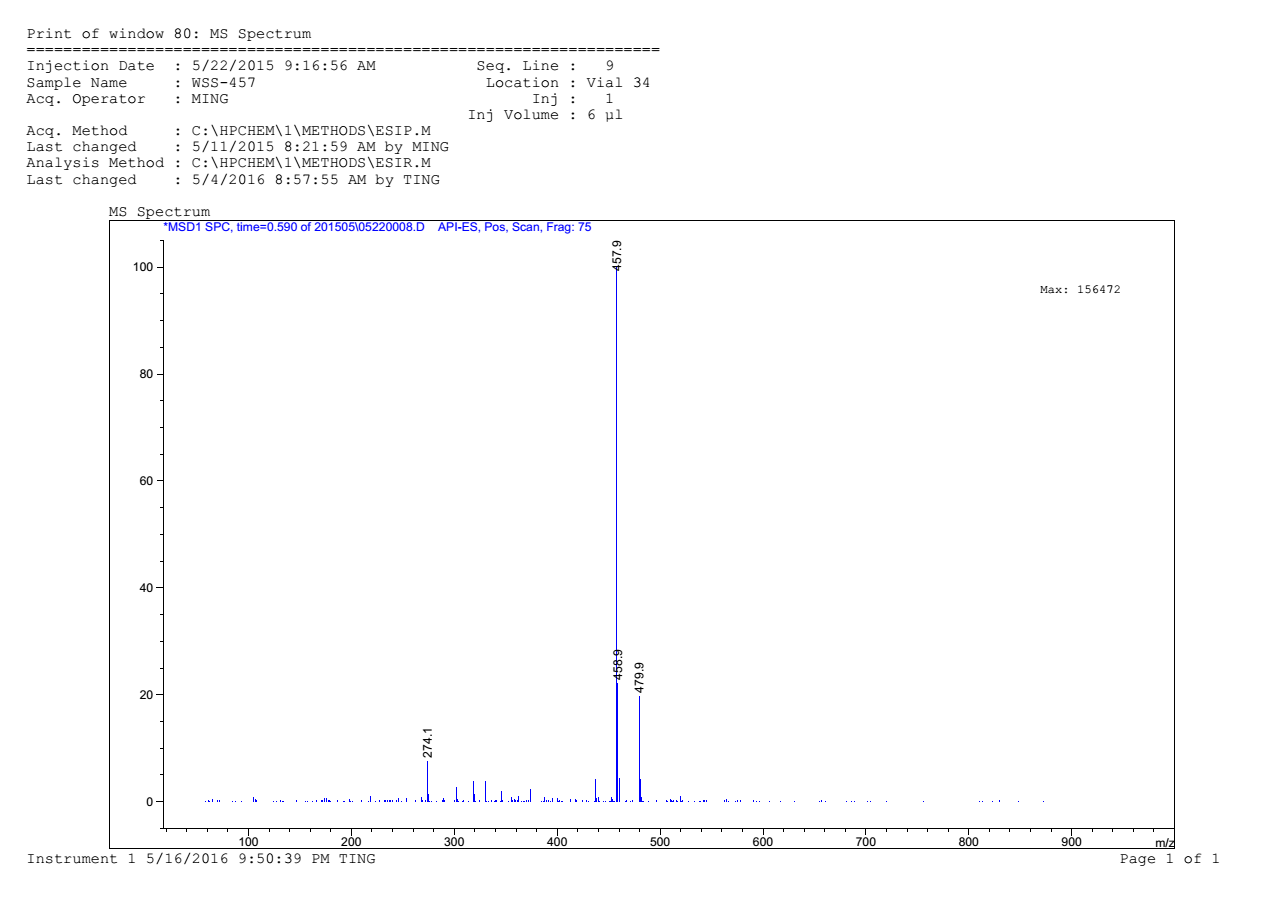
**

**22. 7d**

**
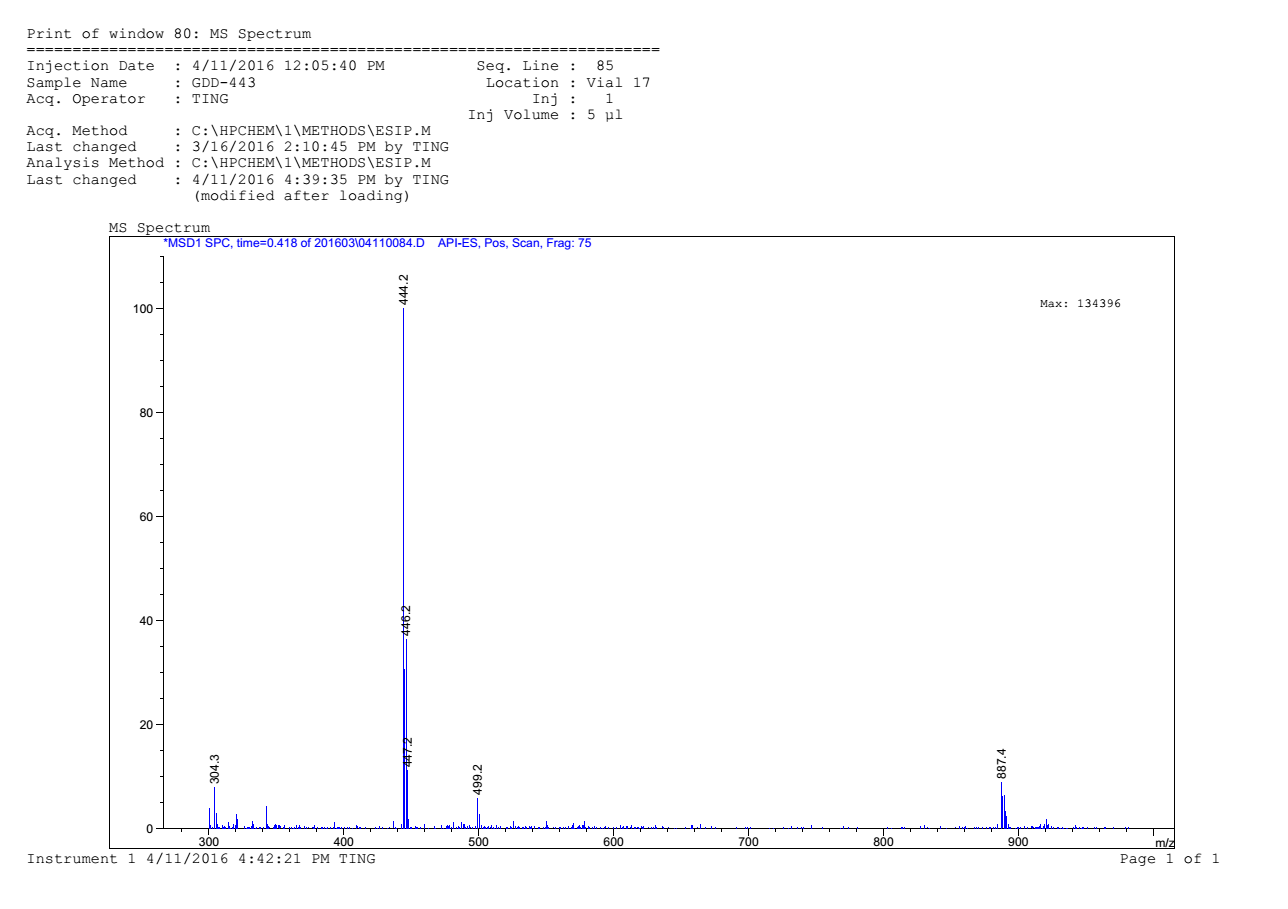
**

**23. 7e**

**
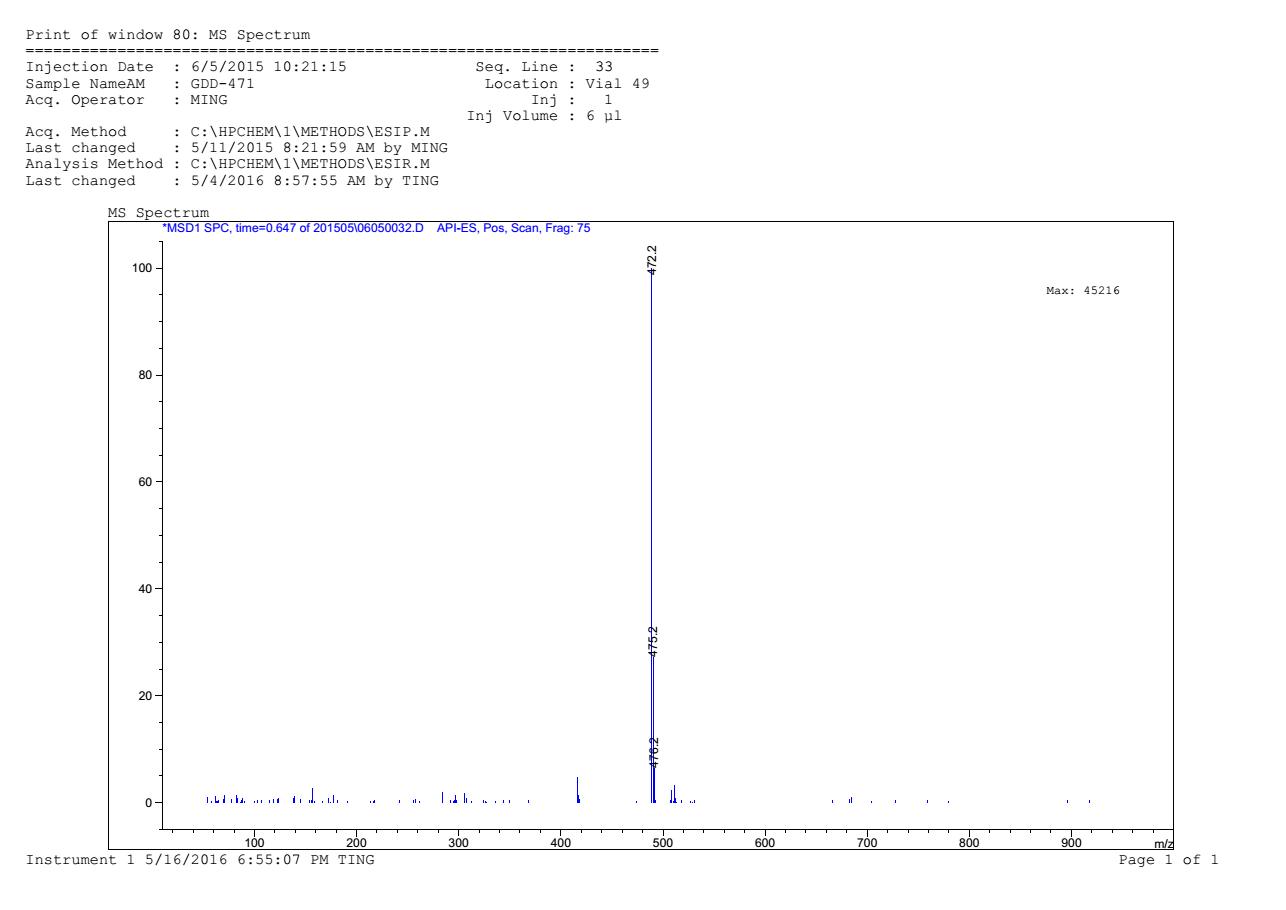
**

**24. 7f**

**
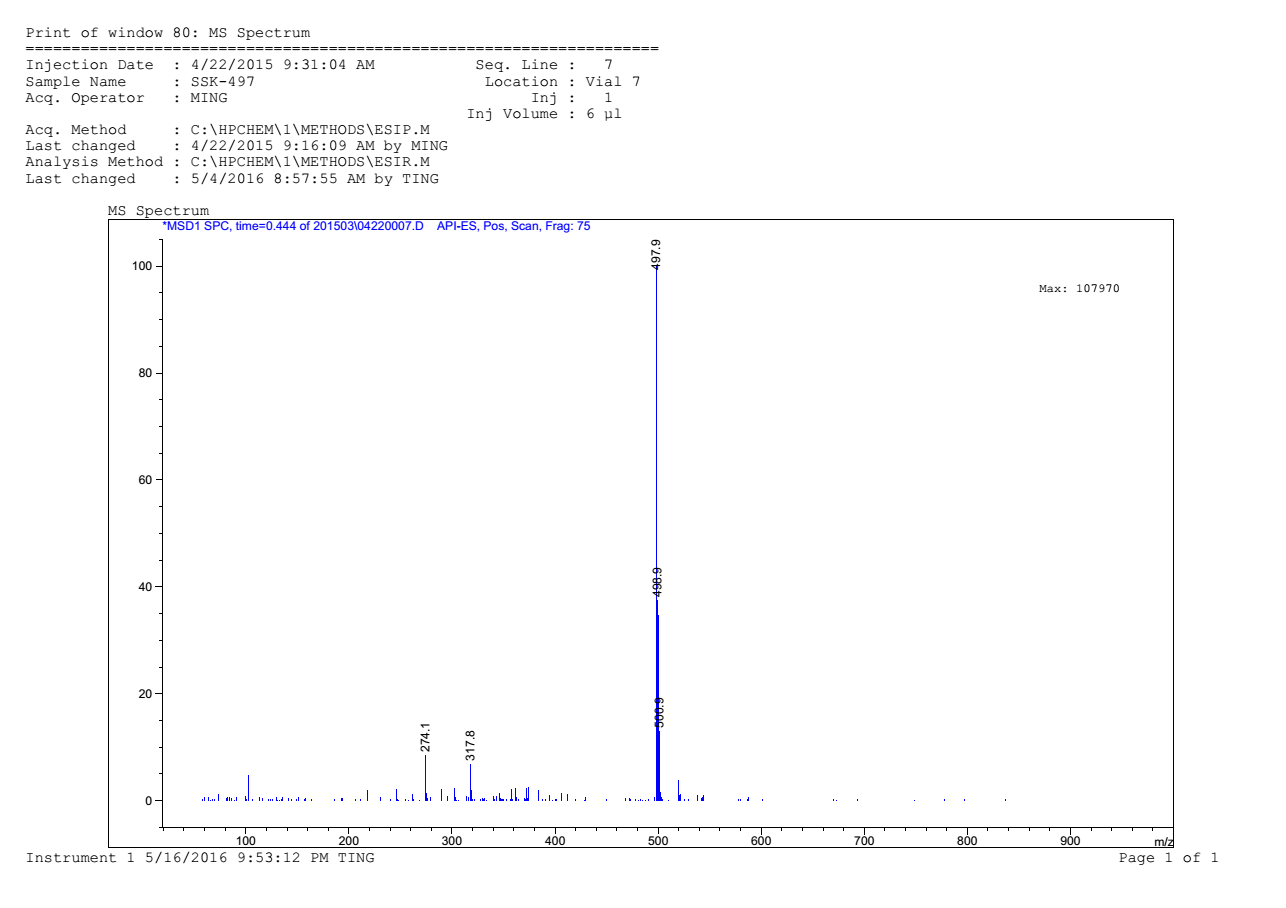
**

**25. 7g**

**
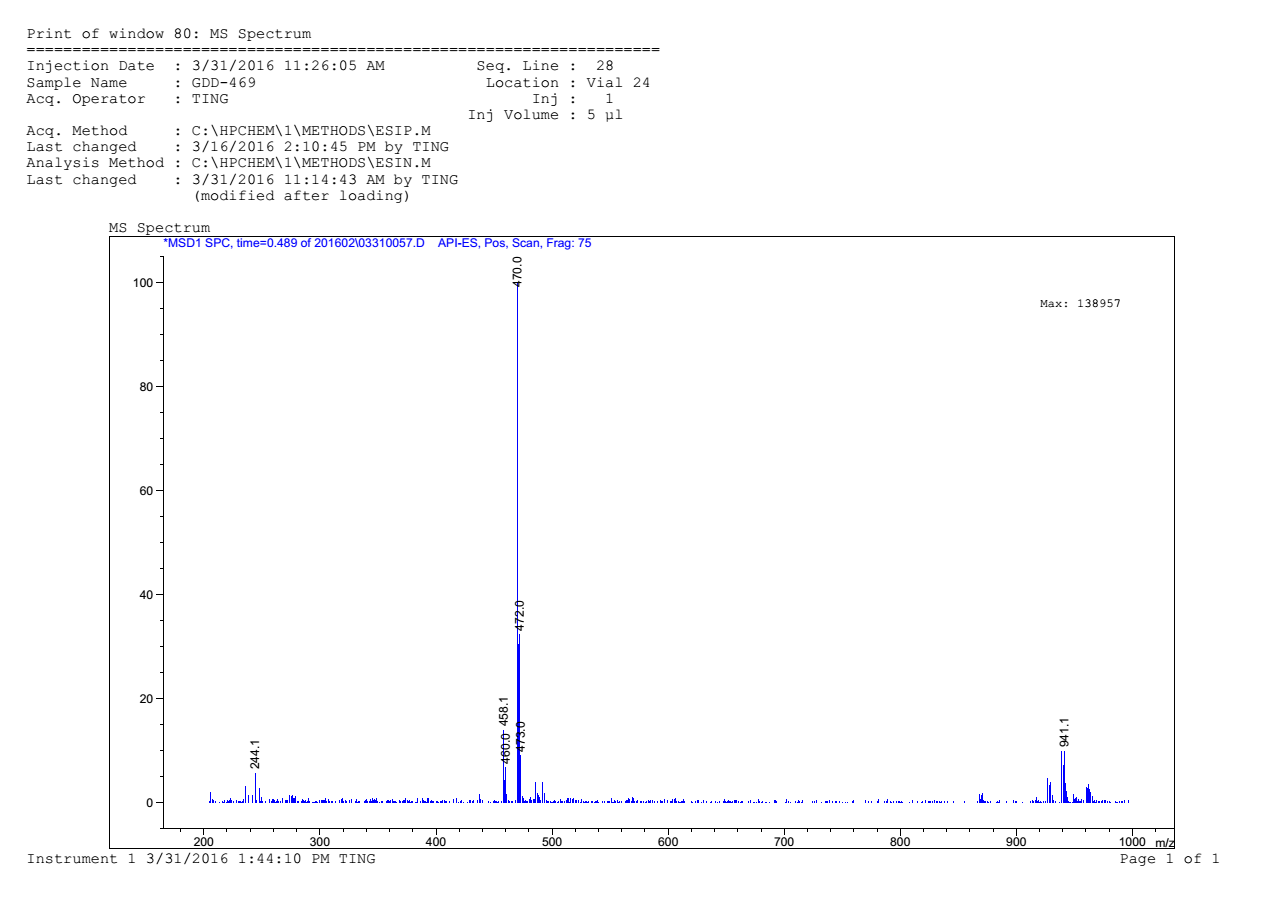
**

**26. 7h**
